# Supplementary material for: Resting natural killer cell homeostasis relies on tryptophan/NAD + metabolism and HIF‐1α
Source: EMBO Rep. 2023 Mar 29;24(6):e56156. doi: 10.15252/embr.202256156 (PMC10240188; doi:10.15252/embr.202256156)
Supplement: Supplementary file 1 — Expanded View Figures PDF [file EMBR-24-e56156-s007.pdf]

Expanded View Figures

Figure EV1. HIF1α is involved in tryptophan/NAD metabolism of splenic NK cells.

A, B NK cell, defined as $CD45^+$ $CD3^-$ $NKp46^+$ $CD49b^+$, (A) count and (B) frequency in bone marrow of WT and HIF-1α KO mice (*n* = 3).
C, D NK cell (A) count and (D) frequency in liver of WT and HIF-1α KO mice (*n* = 3).
E Gene expression analysis of genes from the NAD—tryptophan pathway and key amino acid transporters from bulk RNA-sequencing performed on six samples of freshly isolated NK cells from WT and HIF-1α KO mice.
F The tryptophan pathway (Trp, tryptophan; NFK, N-formylkynurenine; Kyn, kynurenine; KA, kynurenic acid; 3-HK, 3-hydroxykynurenine; 3-HAA, 3-hydroxyanthranilic acid; ACMS, 2-amino-3-carboximuconate semialdehyde; AMS, aminomuconate semialdehyde; QA, quinolinic acid) and GSEA analysis of the RNA expression of its key enzymes (Ido, indoleamine-2,3-dioxygenase; Tdo, tryptophan-2,3-dioxygenase; Afmid, kynurenine formamidase; Kyat, kynurenine aminotransferase; Kmo, kynurenine 3-monooxygenase; Kynu, kynureninase; Haao, 3-hydroxyanthranilate 3,4-dioxygenase; Acmsd, ACMS decarboxylase).
G Analysis of quinolinic acid quantification from targeted metabolomics.
H Analysis of mitochondrial ROS amount of freshly isolated NK cells from bone marrow (BM) and liver of WT and HIF-1α KO mice (*n* = 3).
I Analysis of DNA damage in bone marrow (BM) and liver NK cells from WT and HIF-1α KO mice by FACS measurement of γ-H2AX (*n* = 3).

Data information: Statistical significance was determined by an unpaired Student's *t*-test. Bars represent mean values, error bars indicate the s.e.m., (*n*) represents the number of independent experiments, and each data point represents a biological sample from a mouse.
Source data are available online for this figure.

© 2023 The Authors

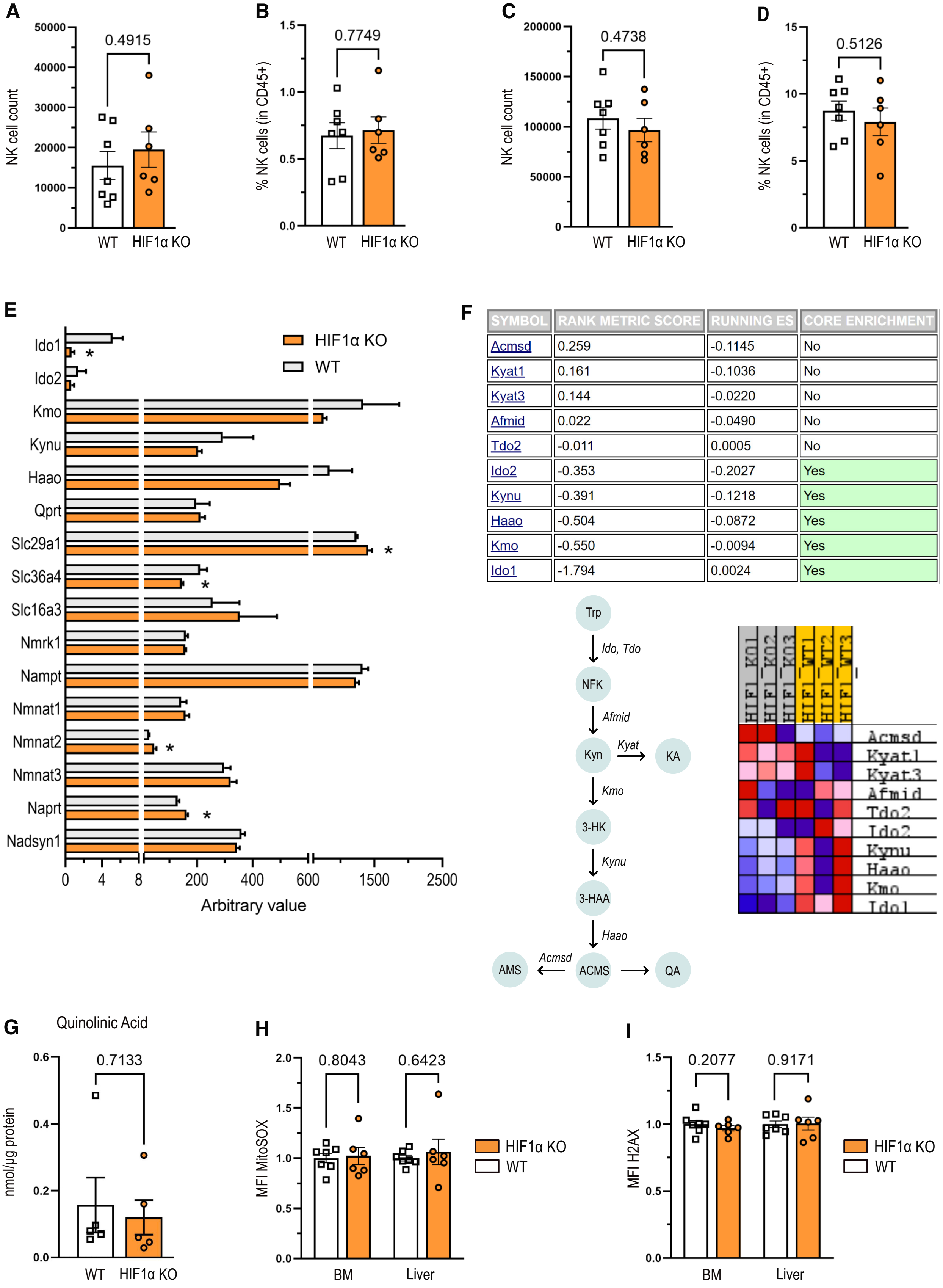

SYMBOL	RANK METRIC SCORE	RUNNING ES	CORE ENRICHMENT
Acmsd	0.259	-0.1145	No
Kyat1	0.161	-0.1036	No
Kyat3	0.144	-0.0220	No
Afmid	0.022	-0.0490	No
Tdo2	-0.011	0.0005	No
Ido2	-0.353	-0.2027	Yes
Kynu	-0.391	-0.1218	Yes
Haao	-0.504	-0.0872	Yes
Kmo	-0.550	-0.0094	Yes
Ido1	-1.794	0.0024	Yes

Figure EV1.

 © 2023 The Authors

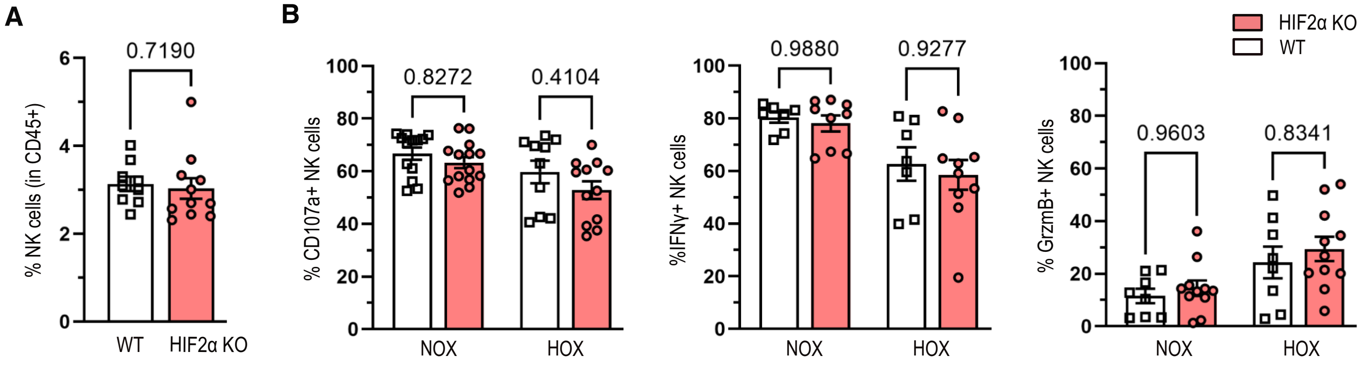

Figure EV2. HIF2α does not play a major role for homeostasis and activation of NK cells.

A NK cell frequency, defined as $CD45^+$ $CD3^-$ $NK1.1^+$ $NKp46^+$, in spleens from WT and HIF-2α KO mice ($n = 3$).

B Splenocytes of WT and HIF-2α KO mice were stimulated with PMA (200 ng/ml) and ionomycin (1 μg/ml) for 6 h at 37°C, 5% CO_2 and 20% O_2 (NOX) or 2% O_2 (HOX) in complete RPMI medium. Degranulation (CD107a) ($n = 3$), IFN-γ ($n = 2$) and granzyme B ($n = 4$) expression were analyzed by flow cytometry.

Data information: Statistical significance was determined by an unpaired Student's *t*-test, one-sample *t*-test or one-way ANOVA where appropriate. Bars represent mean values, error bars indicate the s.e.m., (n) represents the number of independent experiments, and each data point represents a biological sample from a mouse. Source data are available online for this figure.

© 2023 The Authors
