# Supplementary material for: Resting natural killer cell homeostasis relies on tryptophan/NAD + metabolism and HIF‐1α
Source: EMBO Rep. 2023 Mar 29;24(6):e56156. doi: 10.15252/embr.202256156 (PMC10240188; doi:10.15252/embr.202256156)
Supplement: Supplementary file 3 — PDF+ [file EMBR-24-e56156-s005.pdf]

Report

SOURCE DATA TRANSPARENT PROCESS OPEN ACCESS

EMBO reports

Resting natural killer cell homeostasis relies on tryptophan/NAD$^+$ metabolism and HIF-1α

Abigaelle Pelletier[1], Eric Nelius[1], Zheng Fan[1], Ekaterina Khatchatourova[1], Abdiel Alvarado-Diaz[2], Jingyi He[1], Ewelina Krzywinska[1], Michal Sobecki[1], Shunmugam Nagarajan[1], Yann Kerdiles[3], Joachim Fandrey[4], Dagmar Gotthardt[5], Veronika Sexl[6], Katrien de Bock[2] & Christian Stockmann[1,*]

Abstract

Natural killer (NK) cells are forced to cope with different oxygen environments even under resting conditions. The adaptation to low oxygen is regulated by oxygen-sensitive transcription factors, the hypoxia-inducible factors (HIFs). The function of HIFs for NK cell activation and metabolic rewiring remains controversial. Activated NK cells are predominantly glycolytic, but the metabolic programs that ensure the maintenance of resting NK cells are enigmatic. By combining *in situ* metabolomic and transcriptomic analyses in resting murine NK cells, our study defines HIF-1α as a regulator of tryptophan metabolism and cellular nicotinamide adenine dinucleotide (NAD$^+$) levels. The HIF-1α/NAD$^+$ axis prevents ROS production during oxidative phosphorylation (OxPhos) and thereby blocks DNA damage and NK cell apoptosis under steady-state conditions. In contrast, in activated NK cells under hypoxia, HIF-1α is required for glycolysis, and forced HIF-1α expression boosts glycolysis and NK cell performance *in vitro* and *in vivo*. Our data highlight two distinct pathways by which HIF-1α interferes with NK cell metabolism. While HIF-1α-driven glycolysis is essential for NK cell activation, resting NK cell homeostasis relies on HIF-1α-dependent tryptophan/NAD$^+$ metabolism.

Keywords HIF; immunometabolism; natural killer cells; nicotinamide adenine dinucleotide; tryptophan

Subject Categories Cancer; Immunology; Metabolism

DOI 10.15252/embr.202256156 | Received 20 September 2022 | Revised 5 March 2023 | Accepted 10 March 2023 | Published online 29 March 2023

EMBO Reports (2023) 24: e56156

Introduction

Natural killer (NK) cells are a subset of cytotoxic innate lymphoid cells with a unique capacity to kill aberrant cells including virus-infected cells or malignant tumor cells (Trinchieri, 1989; Vivier *et al*, 2004; Spits *et al*, 2016). Upon activation, NK cells release cytotoxic Granzyme B and the cytokine interferon-γ (IFN-γ; Vivier *et al*, 2012; Mace *et al*, 2014). NK cells reside and operate in hypoxic conditions and cellular adaptation to low oxygen is mediated by hypoxia-inducible transcription factors (HIFs), with HIF-1 and HIF-2 being the most extensively studied (Schofield & Ratcliffe, 2004; Kaelin, 2008; Semenza, 2012). HIF-1α has been implicated in the metabolic control of various cell types, especially the use of pyruvate, by inhibiting the entrance of pyruvate into oxidative phosphorylation (OxPhos) to increase its fermentation to lactate (Iyer *et al*, 1998; Papandreou *et al*, 2006; Ullah *et al*, 2006). NK cell activation and effector function largely relies on aerobic glycolysis (Donnelly *et al*, 2014; Marçais *et al*, 2014; Keppel *et al*, 2015; Assmann *et al*, 2017; Isaacson & Mandelboim, 2018; Loftus *et al*, 2018; Poznanski *et al*, 2018), and this switch is controlled by mammalian target of rapamycin complex 1 (mTORC1), Srebp and cMyc (Donnelly *et al*, 2014; Marçais *et al*, 2014; Assmann *et al*, 2017; Loftus *et al*, 2018). In NK cells, HIF-1α is considered as dispensable for glycolysis and the metabolic activation of NK cells (Krzywinska *et al*, 2017; Loftus *et al*, 2018; Ni *et al*, 2020) and the question whether HIF-1α promotes or impairs NK cell effector function is a matter of debate (Krzywinska *et al*, 2017; Ni *et al*, 2020). Taken together, the metabolic programs that ensure homeostasis of resting NK cells in low-oxygen environments are not well understood. Moreover, there are no studies describing a functional role for HIF-1α in resting NK cells. Therefore, we set out to study the impact of HIF-1α on metabolism and homeostasis of NK cells *in vivo*.

Results

The transcription factor HIF1α is involved in tryptophan/NAD metabolism and NK cell homeostasis

In vivo, targeted deletion of HIF-1α was achieved by crosses of the loxP-flanked HIF-1α allele[17] to the *Ncr1* (NKp46) promoter-driven Cre recombinase[17], specific to NKp46-expressing innate lymphoid

1 Institute of Anatomy, University of Zurich, Zurich, Switzerland
2 Department of Health Sciences and Technology, ETH Zurich, Zurich, Switzerland
3 Centre d'Immunologie de Marseille-Luminy, Aix Marseille Université UM2, Inserm, U1104, CNRS UMR7280, Marseille, France
4 Institut für Physiologie, Universitätsklinikum Essen, Universität Duisburg-Essen, Essen, Germany
5 Institute of Pharmacology and Toxicology, University of Veterinary Medicine, Vienna, Austria
6 University of Innsbruck, InnSbruck, Austria
*Corresponding author. Tel: +41 44 63 55300; E-mail: christian.stockmann@anatomy.uzh.ch

© 2023 The Authors. Published under the terms of the CC BY 4.0 license

cells, including NK cells (HIF-1α$^{fl+/fl+}$/Ncr1^{cre+} mice, termed HIF-1α KO). We first analyzed NK cells in organs that harbor a substantial number of NK cells but exhibit different oxygen partial pressures (pO_2), namely the bone marrow (pO_2 = 40–50 mmHg), liver (pO_2 = 30–40 mmHg), and spleen (pO_2 = 20 mmHg; Jagannathan *et al*, 2016). Mice with an NK cell-specific deletion of HIF-1α show reduced NK cell numbers in the spleen and, hence, the organ with the lowest pO_2 (Fig 1A), whereas NK cells from the bone marrow and liver were not affected (Fig EV1A–D). This is in line with previous reports from our laboratory that demonstrated a decrease in splenic NK cells upon loss of HIF-1α, while neither maturation nor the receptor repertoire of NK cells were affected (Krzywinska *et al*, 2017). To understand the underlying mechanism accounting for the perturbed NK cell homeostasis *in situ*, we avoided any metabolic bias introduced by positive selection (binding to activating NK cell receptors), artificial culture conditions (high glucose media) or high-dose cytokine exposure as used during *ex vivo* NK cell expansion/stimulation (Crabtree, 1929). Instead, we captured the impact of HIF-1α on resting splenic NK cells *in situ* with combined untargeted mass spectrometry-based metabolomics and transcriptomic analysis after rapid negative "no touch" selection (purity 90%; Fig 1B). In line with the role of HIF-1α in glycolysis in various cell types, HIF-1α-deficient NK cells exhibit decreased levels of the glycolytic end product lactic acid along with a reduction of the amino acids glutamine, glutamate, proline, and arginine (Fig 1C). Unexpectedly, we observed a drastic decrease in the essential amino acid tryptophan, the downstream tryptophan metabolite methylquinoline as well as the NAD precursor, Niacinamide (NAM), suggesting a role for HIF-1α in tryptophan/NAD metabolism in NK cells under homeostatic conditions. This was further corroborated by reduced mRNA expression of the transporter *Slc36a4* for the amino acids tryptophan, glutamine, and proline and the rate-limiting enzyme in tryptophan catabolism *Indolamin-2,3-Dioxygenase* (*Ido1*) in the absence of HIF-1α (Figs 1D, and EV1E and F). To get further insights, we used targeted metabolomics on the tryptophan/NAD metabolome, which allows to capture more subtle HIF1α-dependent changes. This procedure identified reduced levels of the tryptophan catabolite kynurenine (KYN) as well as the NAD precursors nicotinic acid mononucleotide (NAMN), nicotinic acid riboside (NAR; Fig 1E), and N-methylnicotinamide (MNA), while other metabolites along this pathway remained unchanged (Fig EV1g). While the mRNA expression of enzymes that convert NAD precursors into NAD was not affected (Fig 1D), HIF-1α-deficient cells had a decreased NAD/NADH ratio (Fig 1F). Although the quantification of NAD/NADH ratio here is based on total NAD and NADH, this suggests a so far unanticipated role of HIF-1α in tryptophan metabolism and the maintenance of free NAD^+ levels in resting, nonactivated NK cells.

HIF1α deficiency leads to DNA damage and NK cell death under homeostatic conditions

NAD^+ plays a crucial role in mitochondrial redox homeostasis and DNA damage repair upon oxidative stress (Fang *et al*, 2016; Sedlackova & Korolchuk, 2020). Consistently, HIF-1α-deficient NK cells generate increased mitochondrial reactive oxidative species (ROS) and suffer from increased DNA damage (Mah *et al*, 2010) and cell death (Fig 2A–C). Of note, bone marrow and liver NK cells from HIF1a KO mice do not show elevated ROS levels or increased DNA damage (Fig EV1H and I). ROS formation is frequently associated with proton leakage upon OxPhos in mitochondria (Murphy, 2009). Therefore, we performed real-time measurement of the oxygen consumption rate (OCR) as a measure of mitochondrial respiration with a Seahorse analyzer on splenic NK cells of both genotypes. In line, HIF-1α-deficient NK cells exhibit increased mitochondrial respiration (OCR) and enhanced proton leakage (Fig 2D) as determined by Seahorse assays. A previous study had reported increased OxPhos in HIF1α-deficient NK cells (Ni *et al*, 2020), without analyzing ROS production. Our data and the fact that HIF-1α-deficient NK cells generate less ATP despite increased OxPhos (Fig 2E) suggest an uncoupling of mitochondrial respiration and ATP production.

Radioactive labeling revealed fatty acid oxidation as the major anaplerotic pathway and the preferred carbon source for OxPhos in HIF-1α-deficient NK cells (Fig 2F), whereas glutaminolysis remained unaffected (Fig 2G). Consistently, loss of HIF-1α is associated with enhanced expression of genes involved in fatty acid metabolism (Fig 2H). Of note, increased oxidation of fatty acids in the mitochondria has been shown to be a source of net ROS production (Murphy, 2009).

Consistent with previous reports, loss of HIF-1α results in enhanced ROS production during OxPhos and DNA damage (Kim *et al*, 2006; Zhang *et al*, 2008). However, our data add novel insight and suggest perturbed tryptophan/NAD metabolism and depletion of NAD precursors in HIF1α-deficient NK cells as the underlying mechanism.

In contrast to resting conditions, NK cells engage glycolysis upon activation (Gardiner & Finlay, 2017; Isaacson & Mandelboim, 2018). In line with previous reports, HIF-1α is dispensable for glycolysis in NK cells under normoxic conditions (20% O_2; Loftus *et al*, 2018; Fig 3A). Yet, HIF-1α becomes a central player for glycolysis under hypoxic conditions, when loss of HIF-1α decreases the extracellular acidification rate (ECAR), a surrogate measurement for glycolysis in the Seahorse assay ECAR (Fig 3A). In line with our previous studies (Krzywinska *et al*, 2017), HIF-1α-deficient NK cells show impaired NK cell reactivity *in vitro* as demonstrated by NK cell degranulation ($CD107a^+$) and IFN-γ expression upon activation by PMA/ionomycin under normoxia and hypoxia (Fig 3B). Supplementation of NAD^+ or NAM fails to rescue the defect in NK cell activation (Fig 3C), and it is tempting to speculate that this phenomenon is caused by the anti-glycolytic effect (Fig 3D; Luengo *et al*, 2021). This indicates that resting NK cell homeostasis relies on HIF-1α-dependent tryptophan/NAD metabolism, whereas HIF-1α-driven glycolysis is essential for NK cell activation.

NK cells have a unique capacity to restrict metastatic spread of circulating cancer cells[8]. Therefore, we aimed to determine the impact of HIF-1α expression on NK cell effector function and immunosurveillance of metastatic tumor cells *in vivo*. To this end, we injected syngeneic B16F10 melanoma cells intravenously into HIF-1α KO mice as well as the corresponding WT littermates and analyzed the number of pulmonary metastases 2-week postinjection. Consistent with our *in vitro* observations, loss of HIF-1α in NK cells leads to increased pulmonary metastasis of intravenously injected melanoma cells (Fig 3E). Given the profound effect of HIF1a on NK cell-dependent pulmonary metastasis *in vivo*, we decided to analyze abundance and phenotype of NK cells in lungs from mice after i.v. injection of B16 melanoma cells. As shown in Fig 3F, the increase in pulmonary metastasis HIF1a KO mice is associated with a decrease

 © 2023 The Authors

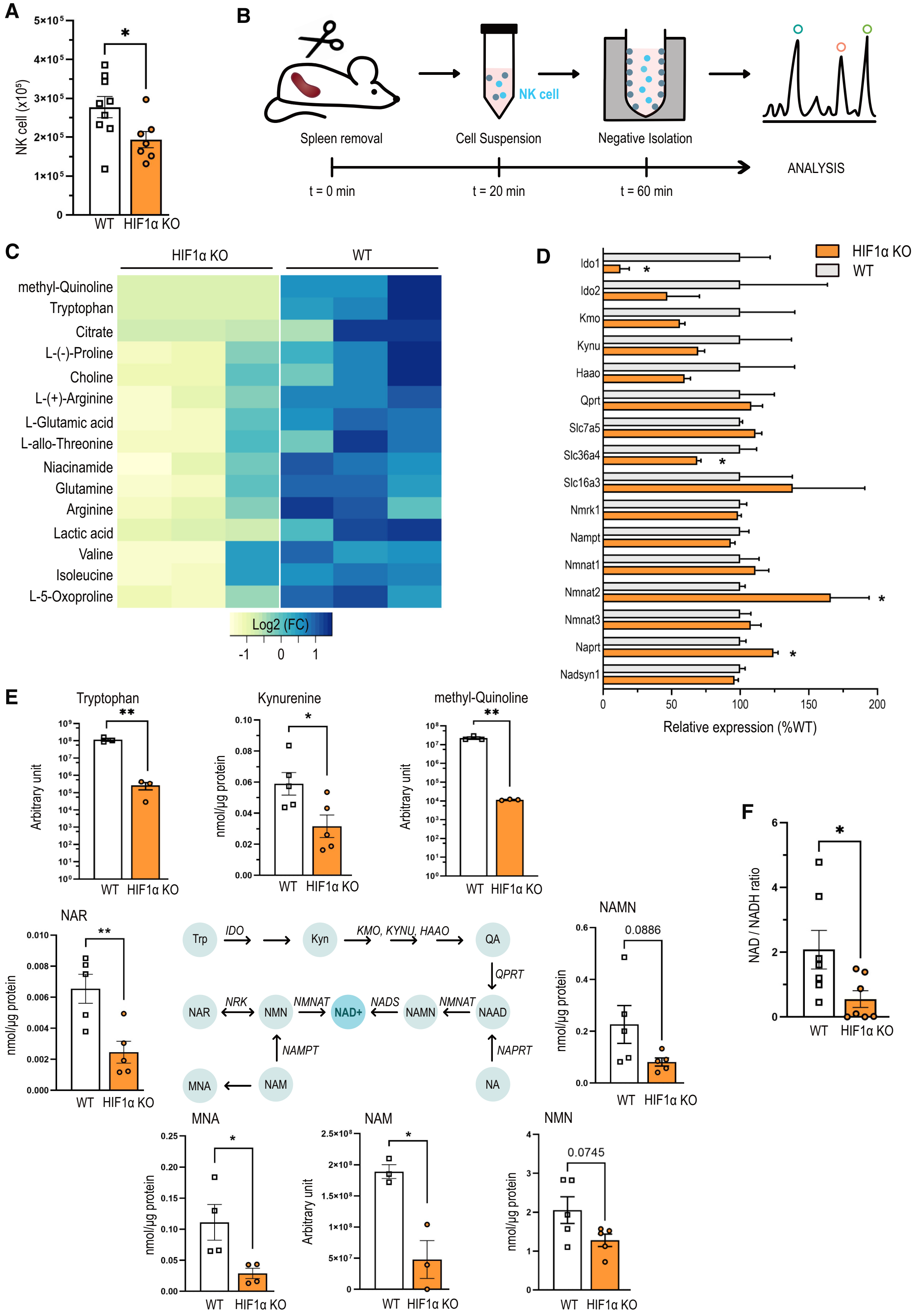

A
NK cell (x10⁵)
WT
HIF1α KO
B
NK cell
Spleen removal
Cell Suspension
Negative Isolation
ANALYSIS
t = 0 min
t = 20 min
t = 60 min
C
HIF1α KO
WT
methyl-Quinoline
Tryptophan
Citrate
L-(-)-Proline
Choline
L-(+)-Arginine
L-Glutamic acid
L-allo-Threonine
Niacinamide
Glutamine
Arginine
Lactic acid
Valine
Isoleucine
L-5-Oxoproline
Log2 (FC)
D
HIF1α KO
WT
Ido1
Ido2
Kmo
Kynu
Haao
Qprt
Slc7a5
Slc36a4
Slc16a3
Nmrk1
Nampt
Nmnat1
Nmnat2
Nmnat3
Naprt
Nadsyn1
Relative expression (%WT)
E
Tryptophan
Kynurenine
methyl-Quinoline
Arbitrary unit
nmol/µg protein
NAR
NAMN
0.0886
Trp
IDO
Kyn
KMO, KYNU, HAAO
QA
QPRT
NAR
NRK
NMN
NMNAT
NAD+
NADS
NAMN
NMNAT
NAAD
NAMPT
NAPRT
MNA
NAM
NA
MNA
NAM
NMN
0.0745
F
NAD / NADH ratio

Figure 1.

Figure 1. The transcription factor HIF1α contributes to tryptophan/NAD metabolism and maintenance of resting NK cells.

A NK cell count, defined as $CD45^+$ $CD3^-$ $NKp46^+$ $CD49b^+$, in spleens of WT and HIF-1α KO mice ($n = 2$).
B Experimental design for *in situ* experiments.
C Heatmap of the downregulated metabolites from the untargeted metabolomics performed on six samples of freshly isolated NK cells from WT and HIF-1α KO mice.
D Gene expression analysis of genes from the NAD—tryptophan pathway and key amino acid transporters from bulk RNA-sequencing performed on six samples of freshly isolated NK cells from WT and HIF-1α KO mice.
E The NAD—tryptophan pathway (Trp, tryptophan; Kyn, kynurenine; QA, quinolinic acid; NAAD, nicotinic acid adenine dinucleotide; NA, nicotinic acid; NAMN, nicotinic acid mononucleotide; NMN, nicotinamide mononucleotide; NAM, nicotinamide; MNA, methylnicotinamide; NAR, nicotinic acid riboside; NAD, nicotinamide adenine dinucleotide) and its key enzymes (IDO, indoleamine-2,3-dioxygenase; KMO, kynurenine 3-monooxygenase; KYNU, kynureninase; HAAO, 3-hydroxyanthranilate 3,4-dioxygenase; QPRT, quinolinate phosphoribosyl-transferase; NMNAT, NMN adenyl-transferase; NAPRT, NA phosphoribosyl-transferase; NADS, NAD synthase; NAMPT, NAM phosphoribosyl-transferase; NRK, NR kinase) and analysis of metabolites quantification from untargeted (tryptophan, methyl-quinoline and NAM) and targeted (NAAD, NA, NMN, MNA and kynurenine) metabolomics.
F NAD/NADH ratio ($n = 1$).

Data information: Statistical significance was determined by an unpaired Student's *t*-test. Bars represent mean values, error bars indicate the s.e.m., (n) represents the number of independent experiments, and each data point represents a biological sample from a mouse (A) or from NK cells pooled from 3 to 5 mice (D–F). Statistical significance is indicated as $^*P < 0.05$, $^{**}P < 0.01$, and $^{***}P < 0.001$.
Source data are available online for this figure.

in pulmonary NK cell frequencies and total numbers, along with a decrease in pulmonary Granzyme B^+ and IFN-γ^+ NK cells. This is consistent with previous reports from our laboratory, where we have shown that loss of HIF1a leads to impaired NK cell infiltration *in vivo* (Krzywinska *et al*, 2017).

Next, we now performed *in vitro* killing assays with B16 melanoma cells with a customized killing assay for B16 cells by increasing the coincubation with NK cells to 24 h and the addition of IL-15 to ensure NK cell survival over this period. However, as shown below, the loss of HIF1a did not affect NK cell-dependent lysis of B16 cells in normoxia or hypoxia (Fig 3G). This is in line with previous reports from our laboratory that HIF1a does not affect the *in vitro* killing of MHC-I-proficient cells (Krzywinska *et al*, 2017) and suggests that HIF-1α is required NK cell infiltration into metastatic lesions.

Constitutive activation of HIF1α enhances glycolysis and NK cell effector function

To explore the functional impact of constitutive HIF activation on NK cells, we created an NK cell-specific deletion of the negative HIF regulator VHL in NK cells, via crosses of the loxP-flanked *Vhl* allele to the *Ncr1* (NKp46) promoter-driven Cre recombinase, ($VHL^{fl+/fl+/}Ncr1^{cre+}$) termed VHL KO (Sobecki *et al*, 2021; Krzywinska *et al*, 2022). NK cell reactivity is strongly linked to NK cell maturation, which can be distinguished by the expression of CD27/CD11b (Hayakawa & Smyth, 2006) along with the development of a repertoire of inhibiting and activating receptors (Huntington *et al*, 2007). Importantly, loss of VHL reduced the frequency and number of immature splenic NK cell frequencies but resulted in a slightly increased frequency of mature NK cells in the spleen (Fig 4A and B), while the receptor repertoire remained unaffected (Fig 4C). Metabolic profiling with the Seahorse analyzer on VHL-deficient splenic NK cells revealed a decreased mitochondrial respiration (OCR) and proton leakage (Fig 4D). Moreover, the absence of VHL enhanced glycolysis (Fig 4E) and NK cell reactivity as demonstrated by increased Granzyme B expression (Fig 4F).

As VHL negatively regulates both HIF isoforms, the deletion of VHL not only stabilizes HIF1α but also extends to the constitutive expression of HIF-2α. To control for these effects, we crossed the loxP-flanked *Epas1* allele[24] that encodes for HIF-2α to the *Ncr1* (NKp46) promoter-driven Cre recombinase[23] ($EPAS1^{fl+/fl+/}Ncr1^{cre+}$, termed HIF-2α KO). In this experimental setting, we failed to detect any effects on splenic NK cell numbers or NK cell activation upon

Figure 2. Loss HIF1α results in oxidative stress and drives apoptosis of resting NK cells.

A Analysis of mitochondrial ROS amount of freshly isolated NK cells from WT and HIF-1α KO mice ($n = 3$).
B Analysis of DNA damage in splenic NK cells from WT and HIF-1α KO mice by FACS measurement of γ-H2AX ($n = 3$).
C Analysis of cell death in freshly isolated NK cells from WT and HIF-1α KO mice using Annexin V and 7AAD staining ($n = 3$).
D Oxygen consumption rate (OCR) analysis of freshly isolated NK cells from WT and HIF-1α KO mice assessed with sequential injection of 1 μM oligomycin, 2 μM FCCP and 1 μM rotenone and antimycin A in normoxia ($n = 8$).
E ATP quantification of freshly isolated NK cells from WT and HIF-1α KO mice ($n = 2$).
F Fatty acid oxidation rate in freshly isolated NK cells from WT and HIF-1α KO mice by measurement of tritium-labeled H_2O release after incorporation of [9,10-^{3}H(N)]-palmitic acid for 6 h ($n = 3$).
G Glutamine oxidation rate in freshly isolated NK cells from WT and HIF-1α KO mice by measurement of ^{14}C-labeled CO_2 release after incorporation of L-[^{14}C(U)]-glutamine for 6 h ($n = 4$).
H GSEA enrichment analysis of upregulated genes in HIF-1α samples from bulk RNA-sequencing performed on three WT samples and three KO samples of freshly isolated NK cells from WT and HIF-1α KO mice.

Data information: Statistical significance was determined by an unpaired Student's *t*-test, one-sample *t*-test or one-way ANOVA where appropriate. Bars represent mean values, error bars indicate the s.e.m., (n) represents the number of independent experiments, and each data point represents a biological sample from a mouse. Statistical significance is indicated as $^*P < 0.05$, $^{**}P < 0.01$, and $^{***}P < 0.001$.
Source data are available online for this figure.

© 2023 The Authors

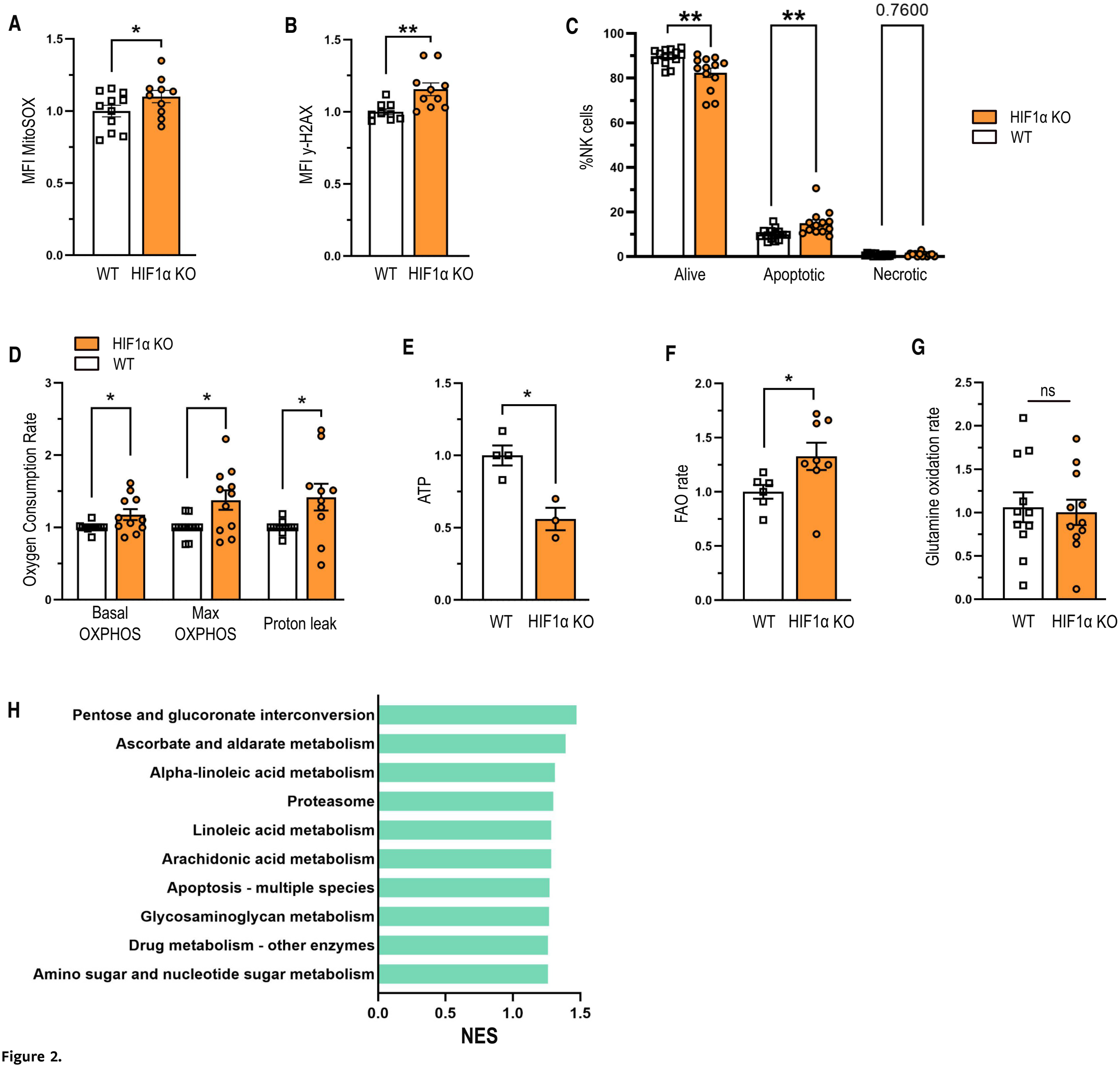

Figure 2.

stimulation PMA/ionomycin under normoxia (20% O_2) and hypoxia (2% O_2) (Fig EV2A and B), suggesting HIF-1α as the main mediator of hypoxic responses in NK cells. However, we did not exclude that a hypomorphic or transdominant negative form of HIF-2 protein may be produced following Cre excision. Moreover, it is important to mention that VHL targets several proteins outside the HIF pathway for ubiquitination and degradation, for example, members of the p53 and NF-κB pathway as well as atypical protein kinase C and RNA polymerase II (Li & Kim, 2011). Therefore, we cannot entirely rule out that loss of VHL also modulates HIF-independent cellular signaling events in NK cells.

Next, we aimed to determine the impact of VHL loss on NK cell immunosurveillance of metastatic tumor cells *in vivo* and injected syngeneic B16F10 melanoma cells intravenously into VHL KO mice as well as the corresponding WT littermates and analyzed the number of pulmonary metastases 2-week postinjection. Consistent with our *in vitro* observations, VHL deletion in NK cells reduced pulmonary metastases by almost 70% (Fig 4G). Furthermore, this was associated with an increase in Granzyme B^+ NK cells, whereas the frequency of total NK cells remained unchanged (Fig 4H). This suggests that forced HIF-1α-driven glycolysis can enhance NK cell effector function *in vivo*.

© 2023 The Authors

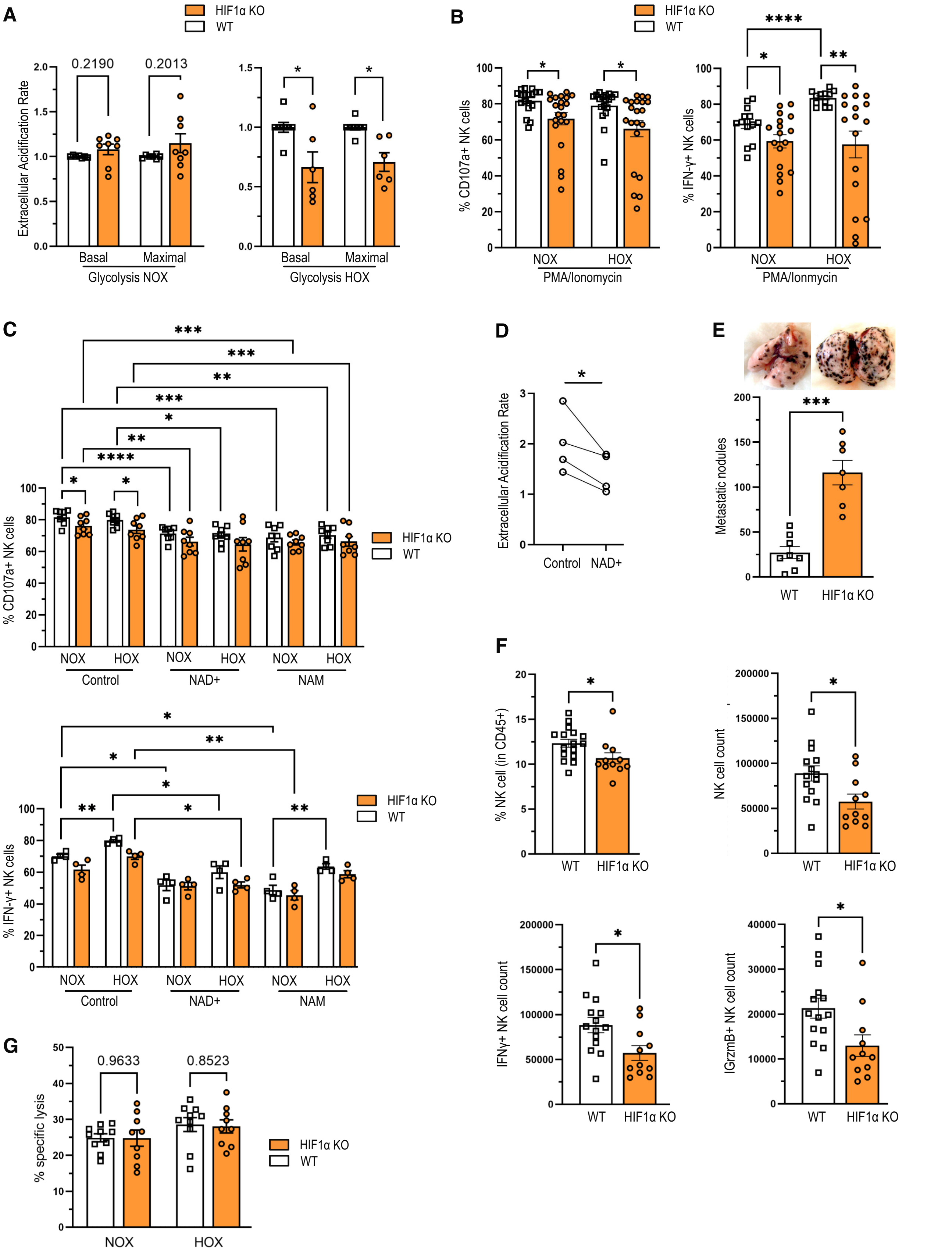

Figure 3.

 © 2023 The Authors

Figure 3. HIF1α is required for glycolysis and full NK cell effector function.

A Extracellular acidification rate (ECAR) analysis of freshly isolated NK cells from WT and HIF-1α KO mice assessed with sequential injection of 10 mM glucose, 1 μM oligomycin and 30 mM 2-deoxy glucose in 20% O_2 (NOX) or 1% O_2 (HOX) (*n* = 6).
B NK cell degranulation ($CD107a^+$) and IFN-γ expression were analyzed by flow cytometry. Splenocytes of WT and HIF-1α KO mice were stimulated with PMA (200 ng/ml) and ionomycin (1 μg/ml), control being unstimulated splenocytes, for 6 h at 37°C, 5% CO_2 and 20% O_2 (NOX) or 2% O_2 (HOX) (*n* = 4).
C Same as (B) compared with supplementation with 10 mM NAD^+ or NAM (*n* = 2).
D ECAR analysis of freshly isolated NK cells from WT and HIF-1α KO mice assessed with sequential injection of 10 mM glucose, 1 μM oligomycin and 30 mM 2-deoxy glucose in normoxia (*n* = 1).
E Pulmonary metastatic nodules count 14 days after intravenous injection of B16F10 tumor cells in WT and HIF-1α KO mice.
F Frequency and count of total infiltrated NK cells, and count of IFN-γ^+ and granzyme B (GrzmB) positive NK cells in metastatic lungs of WT and HIF-1α KO mice (*n* = 3).

Data information: Statistical significance was determined by an unpaired Student's *t*-test, one-sample *t*-test or one-way ANOVA where appropriate. Bars represent mean values, error bars indicate the s.e.m., (*n*) represents the number of independent experiments, and each data point represents a biological sample from a mouse. Statistical significance is indicated as $*P < 0.05$, $**P < 0.01$, and $***P < 0.001$.
Source data are available online for this figure.

Discussion

In summary, this study suggests that forced HIF-1α-driven glycolysis can enhance NK cell effector function, whereas the homeostasis of resting NK cell homeostasis relies on HIF-1α-dependent tryptophan/NAD metabolism. Nicotinamide adenine dinucleotide (NAD) is an important cofactor in metabolic reactions. Its oxidized form, NAD^+, can accept electrons from glycolysis and Krebs cycle's reactions, while its reduced form releases electrons in the electron transport chain, leading to the production of ATP. It has been shown that there is a tight interplay between ATP production and the balance between NAD^+ and NADH. Indeed, aerobic glycolysis engaged in proliferative cells such as activated NK cells is not only need for ATP production but also for NAD^+ regeneration, and the need of NAD^+ over ATP leads to a metabolic switch to aerobic glycolysis (Luengo *et al*, 2021). Thus, the metabolic shift induced by HIF-1α is likely to influence the NAD^+/NADH balance in cells. However, this question had not been studied yet. Our data indeed suggest a role for HIF-1α in the maintenance of free NAD^+ levels in resting, nonactivated NK cells.

The question whether HIF-1α promotes or impairs NK cell effector function has been controversial (Krzywinska *et al*, 2017; Ni *et al*, 2020). Although we cannot exhaustively clarify all the contradictions between our studies and the report by Ni *et al*, who report enhanced NK cell function upon loss of HIF-1α, we would like to propose some explanations for the obvious discrepancies. One likely explanation is the use of different tumor models and, hence, target cells. We used in this and previous studies YAC-1 and v-ABL lymphoma cell lines as well as LLC lung carcinoma, MC 38 colon cancer, and B16F10 melanoma cell lines for *in vivo* studies. Ni *et al* mostly studied subcutaneous RMA-s and RMA tumors and in one experiment LLC tumors with different initial numbers of subcutaneously injected tumor cells. Of note, Ni *et al* did not perform intravenous injection of B16 cells as we did here, to selective study the impact of HIF1α on NK cell surveillance of circulating melanoma cells. Additional confounding factors could be the degree of backcrossing to the BL6 background (in our case > 99% C57Bl/6J background) as well as varying hygiene levels in different animal husbandry and the potential consequences on preactivation of NK cells.

Besides that, Ni *et al* performed all *ex vivo* Seahorse analysis under normoxic conditions and not in hypoxia. Yet, Ni *et al* also observe increased OxPhos in HIF1α deficient NK cells in Seahorse. However, whether increased OxPhos affects ROS production or DNA damage was not evaluated further in the study by Ni *et al*.

Finally, in contrast to our studies, Ni *et al* did not observe significant differences in splenic NK cell numbers. Although there was a trend toward decreased NK cell numbers in HIF1α KO mice with *n* = 3, this trend failed to reach the level of statistical significance. Although we cannot entirely reconcile our results with the report by Ni *et al*, the above-mentioned differences might explain some of the contradictions.

Figure 4. Forced HIF1α expression enhances NK cell glycolysis and effector potential.

A NK cell frequency and count, defined as $CD45^+$ $CD3^-$ $NK1.1^+$ $NKp46^+$, in spleens from WT and VHL KO mice (*n* = 3).
B, C Expression analysis of (B) maturation markers and (C) inhibitory and activating receptors of freshly isolated NK cells from WT and VHL KO mice (*n* = 2).
D OCR analysis of freshly isolated NK cells from WT and VHL KO mice assessed with sequential injection of 1 μM oligomycin, 2 μM FCCP and 1 μM rotenone and antimycin A in normoxia (*n* = 10).
E ECAR analysis of freshly isolated NK cells from WT and VHL KO mice assessed with sequential injection of 10 mM glucose, 1 μM oligomycin, and 30 mM 2-deoxy glucose in 20% O_2 (NOX) (*n* = 12) or 1% O_2 (HOX; *n* = 8).
F Splenocytes of WT and VHL KO mice were stimulated with PMA (200 ng/ml) and ionomycin (1 μg/ml) for 6 h at 37°C, 5% CO_2 and 20% O_2 (NOX) or 2% O_2 (HOX) in complete RPMI medium. Granzyme B expression was analyzed by flow cytometry (*n* = 2).
G Pulmonary metastatic nodules count 14 days after intravenous injection of B16F10 tumor cells in WT and VHL KO mice.
H Frequency of total and granzyme B positive ($GrzmB^+$) infiltrated NK cell in metastatic lungs of WT and VHL KO mice (*n* = 1).

Data information: Statistical significance was determined by an unpaired Student's *t*-test, one-sample *t*-test or one-way ANOVA where appropriate. Bars represent mean values, error bars indicate the s.e.m., (*n*) represents the number of independent experiments, and each data point represents a biological sample from a mouse. Statistical significance is indicated as $*P < 0.05$, $**P < 0.01$, $***P < 0.001$, and $****P < 0.0001$.
Source data are available online for this figure.

© 2023 The Authors

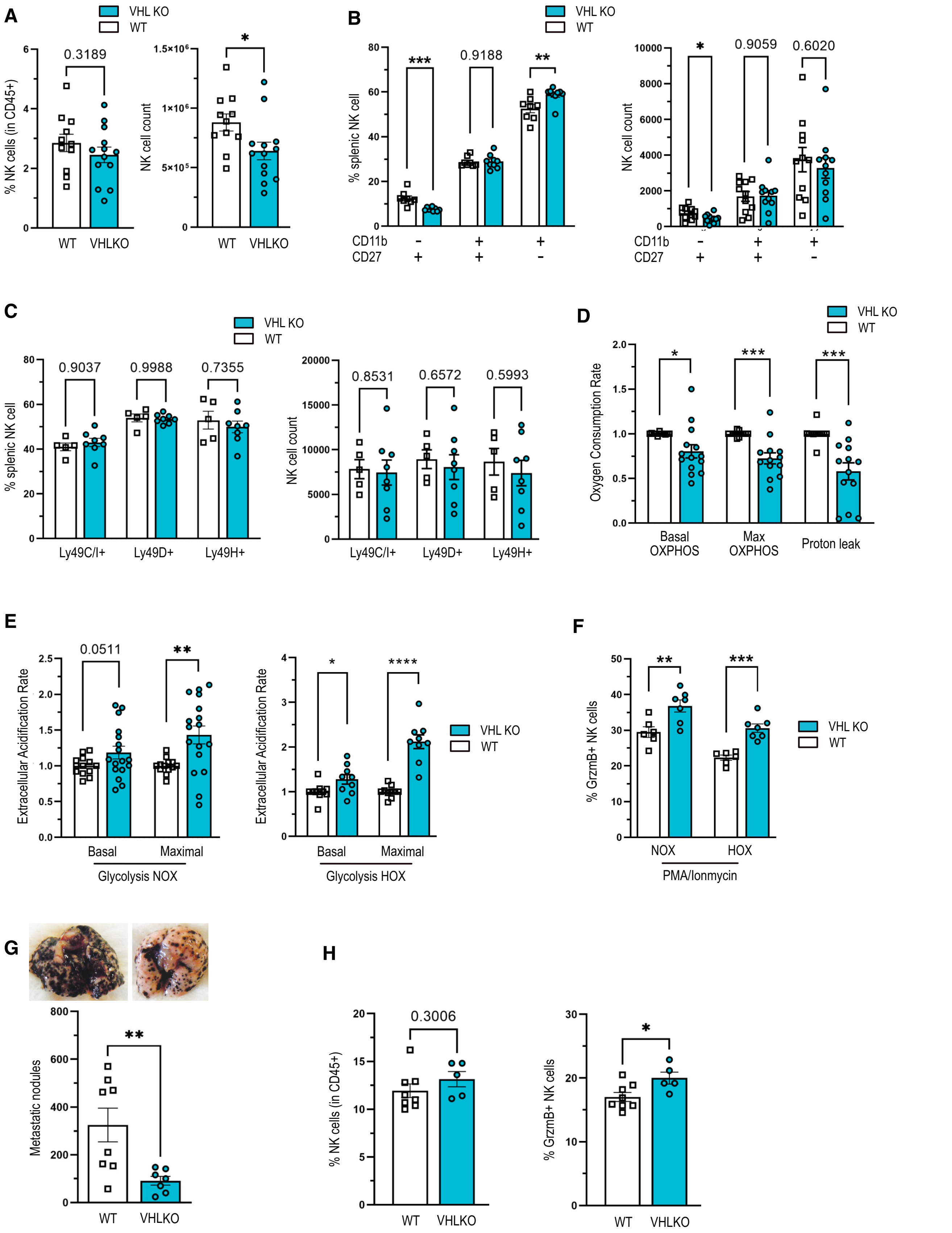

Figure 4.

© 2023 The Authors

In summary, we demonstrate that forced HIF-1α-driven glycolysis enhances NK cell effector function and define a novel role of HIF-1α in the tryptophan/NAD pathway to ensure redox homeostasis of resting NK cells.

Materials and Methods

Mouse models

In vivo targeted deletions of HIF-1α and VHL in NK cells were achieved by as previously described (Krzywinska *et al*, 2017, 2022). To mitigate the influence of strain variation, mice were kept in a > 99% C57Bl/6J background. Male and female mice at the age of 8–12 weeks were used at equal ratios. All animal experiments were approved by the local animal ethics committee (Kantonales Veterinärsamt Zürich, licenses ZH208/18 and ZH189/18) and performed according to local guidelines (TschV, Zurich) and the Swiss animal protection law (TschG).

Pulmonary metastasis model

B16F10 melanoma cell line was cultured in a RPMI GlutaMAX TM medium (Thermo Fisher) supplemented with 10% of fetal bovine serum (FBS) and 50 U/ml penicillin–streptomycin at 37°C and 5% CO_2. Cells were resuspended in PBS and 1×10^5 (for HIF1α mouse model) or 2×10^5 (for VHL mouse model) B16F10 cells in 50 μl of PBS were injected into the tail vein of the animals. The lungs were removed after 7 days for NK cell infiltration assessment and stimulation assay and after 14 days for metastatic foci counting.

NK cell purification

Splenocytes, bone marrow and liver cell suspensions were obtained from HIF-1α WT and KO mice. NK cells from spleen and bone marrow were purified on ice using "EasySep™ Mouse NK Cell Isolation Kit" following the manufacturer's protocol. NK cells from liver were purified as described previously (Krzywinska *et al*, 2017).

Stimulation assay

Splenocytes were prepared and cultured with PMA (20 ng/ml) and ionomycin (1 μg/ml) in a complete RPMI medium for 6 h at 37°C in normoxia (20% O_2) or hypoxia (1% O_2). Golgi Stop (BD), Golgi Plug (BD) and anti-CD107a were added in the medium at the beginning of stimulation. Surface and intracellular staining for granzyme B and INF-γ was performed.

Killing assay

B16F10 melanoma cells were stained with CellTrace Violet (Thermo Fisher) following the manufacturer's protocol prior to the assay. Freshly isolated NK cells were co-cultured at a ratio of 10:1 (NK:target) with B16F10 target cells for 24 h in a complete RPMI medium in normoxia (20% O_2) or hypoxia (2% O_2). After 24 h, cells were trypsinized for 4 min and acquired by FACS. Sytox Green (Thermo Fisher) was added in each FACS tube 2 min prior to acquisition.

Flow cytometry

Flow cytometry was carried out on a LSR II Fortessa or a FACSymphony (BD Bioscience) with anti-phospho-Histone H2A.X Monoclonal (MA537049, Thermo Fisher), anti-CD45 (1033132, BioLegend), anti-CD3 (100222, BioLegend), anti-NK1.1 (108748, Biolegend), anti-NKp46 (137606, BioLegend), anti-CD107a (560648, BD Biosciences), anti-Granzyme B (812-8898, Invitrogen), anti-Interferon-gamma (554413, BD), and Annexin V and 7AAD (BioLegend, 640934).

Seahorse metabolic flux analysis

Freshly isolated NK cells were seeded at 200,000 cells/ well either in an 8-well or in a 96-well Seahorse plate in 180 μl of the Seahorse RPMI medium supplemented with 10 mM glucose, 2 mM sodium pyruvate, and 2 mM L-glutamine. For real-time measurement of oxygen consumption rate (OCR), sequential injection of 1 μM oligomycin, 2 μM FCCP and 1 μM of rotenone and antimycin A was performed. For real-time measurement of extracellular acidification rate (ECAR), sequential injection of 10 mM glucose, 1 μM oligomycin and 30 mM of 2-deoxy glucose (2-DG) was performed. Seahorse culture microplate was coated with Cell-Tak to make NK to adhere to the plate. For the measurement of ECAR in hypoxia, NK cells were preincubated in a non-CO_2 incubator at 1% O_2 for 1 h prior measurement under hypoxia.

ATP quantification

ATP levels were quantified using "ATP Determination Kit" (Thermo Fisher) on freshly isolated NK cells following the manufacturer's instructions. Briefly, 200,000 freshly isolated NK cells were plated in a V-bottom 96-well plate. A standard curve for ATP, ranged from 5 nM to 5 μM, was prepared. In each well, 90 μl of standard reaction solution was added to the sample or standards and incubated for 15 min on the benchtop. Finally, luminescence was measured using a 560 nm filter.

ROS quantification

Mitochondrial ROS were measured using MitoSOX Red™ reagent (Thermo Fisher, Invitrogen). NK cells were plated in a V-bottom 96-well plate in 200 μl of 5 μM MitoSOX solution and incubated for 10 min at 37°C, 20% O_2 and 5% CO_2. Cells were incubated with Aqua Live/Dead at 1/500 for 20 min at 37°C, 20% O_2 and 5% CO_2. Cells were resuspended in FACS buffer, and the mean fluorescence intensity (MFI) was acquired by flow cytometry with an LSR Fortessa (BD Biosciences) or FACSymphony (BD Bioscience).

Glutamine oxidation rate

Glutamine oxidation was measured with freshly isolated NK cells. The protocol was adapted from Veys *et al* (2019). On the first day, NK cells were isolated and resuspended in a labeling solution of RPMI Glutamax + penicillin/streptomycin + 5 μCi/ml L-[^{14}C(U)]-glutamine. In each well, 50 μl of radioactive solution was added to 500,000 NK cells in triplicate. To avoid any contamination, triplicates of each mouse were plated alone in one plate and not mixed

© 2023 The Authors

with other mouse replicates. The columns 1 and 12 and rows A and H of the plates were not used and filled with 200 µl of fresh PBS. Plates were incubated for 6 h at 37°C, 5% CO_2 and 20% O_2. In the meantime, filter papers of 2.5 × 2.5 cm were prepared for the next step of the protocol. A few minutes before the end of the incubation, a 1× hyamine solution was made with fresh pure water and the filter papers were soaked in the solution. After incubation, 12.5 µl of 3 M perchloric acid was added in each well and a soaked filter paper was quickly added on top of the well. The hyamine surplus of each filter paper was carefully removed by skimming the filter on a tissue. The plate was then sealed with tape and incubated for 16 h on the benchtop.

On the second day, scintillation vials were filled with 5 ml of scintillation liquid. At the end of the incubation, the seal of each plate was carefully removed to avoid any spill and each filter paper was transferred into a scintillation vial. For the standards, 2 and 4 µl of labeling solution were added in duplicate in empty scintillation vials. Vials were shaken thoroughly, and filter papers were allowed to disperse their radioactive material into the scintillation liquid for 24 h in the dark at room temperature. After 24 h, the disintegration per minute (DPM) was measured for each vial using the protocol for ^{14}C measurement for 5 min.

Fatty acid oxidation rate

Fatty acid oxidation was measured with freshly isolated NK cells. The protocol was adapted from (Veys *et al*, 2019). On the first day, NK cells were isolated and resuspended in a labeling solution composed of RPMI Glutamax + penicillin/streptomycin +20 µCi/ml [9,10-^3H (N)]-palmitic acid + 100 µM palmitate bound to fatty acid-free BSA + 500 µM carnitine. In each well, 50 µl of radioactive solution was added to 300,000 NK cells in triplicate. The plate was sealed with a plastic film allowing gas exchange but enabling water loss. It was then incubated for 6 h at 37°C, 5% CO_2 and 20% O_2. In the meantime, rubber stoppers with hanging wells were prepared. In brief, a filter paper of 1 × 6 cm was rolled and inserted in a hanging well. The hanging well was then inserted in a rubber cap of a glass vial. The same was prepared for each well of the 96-well plate. Just before the end of the incubation time, each filter paper was hydrated by adding 200 µl of fresh ultrapure water in the hanging well. At the end of the incubation, the 50 µl of the labeling solution was transferred into a glass vial. Then, 12.5 µl of 3 M perchloric acid was added to each vial to stop the metabolic activity of unintentionally transferred cells. Vials were quickly closed with a rubber cap containing the hanging well to avoid any loss of radioactive water. The vials were then incubated at 37°C, 5% CO_2 and 20% O_2 for 48 h.

On the third day, at the end of the incubation, scintillation vials were filled with 5 ml of scintillation liquid. Each glass vial was carefully opened to avoid any spill, and the filter paper was transferred into the scintillation vial. For the standards, 2 and 4 µl of labeling solution were added in duplicate in empty scintillation vials. Vials were shaken thoroughly, and filter papers were allowed to disperse their radioactive material into the scintillation liquid for 24 h in the dark at room temperature. The glassware material, the rubber stopper, and the plastic hanging well were carefully washed with plenty of water and soap. After 24 h, the disintegration per minute (DPM) was measured for each vial using the protocol for ^3H measurement for 5 min.

NAD quantification

NAD^+ and NADH were extracted from freshly isolated NK cells, and total NAD and NADH were quantified using NAD/NADH Quantification Kit (Sigma-Aldrich). NK cells were isolated and 1 million NK cells were pelleted; the supernatant was removed, and 400 µl of NADH/NAD extraction buffer was added. Extraction was performed by two cycles of 20 min freezing on dry ice followed by 15 min of thawing at room temperature. Samples were then vortexed for 10 s, transferred in 10 kDa cutoff spin filters, and centrifuged for 10 min at 14,000 *g*. Half of the extract was kept in the fridge until use, and half of the extract was heated for 30 min at 60°C to decompose NAD^+. A standard curve for NADH was made ranging from 0 to 20 pmoles. A master mix, composed of NAD cycling buffer and NAD cycling enzyme mix, was freshly prepared as described in the kit's protocol. For each sample, 50 µl of total NAD extract and NADH (decomposed) extract was plated in duplicate in a clear 96-well flat bottom plate, and standards were also plated in duplicate. In each well, 100 µl of master mix was added and mixed well by pipetting. The plate was incubated for 5 min on the benchtop. Finally, 10 µl of NADH Developer was added to each well and the plate was incubated for 2 h at room temperature. At the end of the incubation, absorbance at 450 nm was measured using a Cytation 5.

Apoptosis

NK cells were isolated and resuspended in 100 µl of ice-cold Annexin V binding buffer. Cells were incubated for 15 min on ice. The buffer was then removed, and cells were resuspended in 50 µl of cold antibodies at 1/100 in Annexin buffer. After 30 min of incubation at 4°C, 200 µl of ice-cold 1× Annexin V binding buffer was added on top to dilute the antibodies. Cells were resuspended in 100 µl of ice-cold 1× Annexin V binding buffer and acquired on the FACSymphony.

Metabolomics

Freshly isolated NK cells from 20 mice were pooled for the untargeted metabolomics, and NK cells from two mice were pooled for the targeted metabolomics. Once isolated, NK cells were washed once in cold PBS and transferred in a cryotube. After centrifugation, the supernatant was removed, and the cell pellet was snap frozen in liquid nitrogen. Samples were kept at −80°C until they were sent to Creative Proteomics who performed the sample preparation and the metabolomics experimental workflow.

RNA isolation and transcriptomic analysis

RNA was isolated from freshly isolated NK cells using RNeasy Mini Kit® (Qiagen). In this experiment, spleens of 2 to 3 mice were processed together. NK cell pellet was resuspended in 350 µl of buffer RLT supplemented with 1% of β-mercaptoethanol. The suspension was mixed by pipetting to help cell, and ethanol 70% was added to each tube which was transferred to a RNeasy spin column. The tube was then centrifuged for 15 s at 10,000 *g*. The flow-through was then discarded, and 350 µl of buffer RW1 was added to the column. The tube was centrifuged at 10,000 *g* for 15 s, and the flow-through was discarded. DNase I solution (30 Kunitz units) was

 © 2023 The Authors

added on the membrane of the spin column and placed at room temperature for 15 min. The same amount of buffer RW1 was added to the column, and the tube was centrifuged for 15 s at 10,000 *g*. Twice, 500 µl of the buffer RPE was added to the tube and centrifuged for 15 s at 10,000 *g*. The column was dried by another round of centrifugation at full speed for 1 min using a new collection tube. Finally, the column was placed on a 1.5 ml collection tube and 30 µl of RNase-free water was added to the column and was centrifuged for 1 min at 10,000 *g*. The tube containing the RNA was then snapped frozen in liquid nitrogen and stored at −80°C until shipment. RNA-sequencing was performed on pooled splenic NK cells of 2–3 mice per sample by Novogene Europe.

Statistical analysis

Data are represented as mean values and standard errors of the mean (SEM). For each chart, the number of independent experiments (n) is given in the legend of the figure. Statistical analysis was performed with GraphPad Prism software 9.1. Statistical significance was evaluated using the one-sample t-test, which compares the mean of the sample with a hypothetical mean, or the unpaired Student's t-test when appropriate. The Gaussian distribution of the values was confirmed for each group before running any test where this hypothesis is assumed. Statistical significance is indicated as $^{*}P < 0.05$, $^{**}P < 0.01$, $^{***}P < 0.001$, and $^{****}P < 0.0001$.

Data availability

The datasets produced in this study are available in the following databases:

- RNA-Seq data: ArrayExpress E-MTAB-12082 (https://www.ebi.ac.uk/biostudies/arrayexpress/studies/E-MTAB-12082).
- Targeted Metabolomics: Metabolights MTBLS6863 (https://www.ebi.ac.uk/metabolights/MTBLS6863/descriptors).

Expanded View for this article is available online.

Acknowledgements

We appreciate the support of the Swiss National Fund (310030_179235 and 310030_212504), the Swiss Cancer League (KFS-4398-02-2018 and KFS-5402-08-2021) and the Swiss National Centre for Competence in Research "Kidney.CH" (N-403-06-26 HCP project grant) to CS. MS was supported by the internal postdoc funding program of the University of Zurich (Forschungskredit UZH Postdoc 2019). We thank the Laboratory Animal Services Center (LASC) for animal care and the UZH Irchel Flow Cytometry core facility. We acknowledge the support from the SKINTEGRITY.CH collaborative research program. Open access funding provided by Universitat Zurich.

Author contributions

Abigaelle Pelletier: Software; formal analysis; investigation; methodology; writing – original draft; project administration; writing – review and editing. **Eric Nelius:** Formal analysis; investigation. **Zheng Fan:** Investigation. **Ekaterina Khatchatourova:** Formal analysis; investigation. **Abdiel Alvarado-Diaz:** Investigation; methodology. **Jingyi He:** Investigation. **Ewelina Krzywinska:** Formal analysis; investigation; methodology. **Michal Sobecki:** Formal analysis; investigation; methodology. **Shunmugam Nagarajan:** Formal analysis; investigation. **Yann Kerdiles:** Validation. **Joachim Fandrey:** Validation. **Dagmar Gotthardt:** Validation. **Veronika Sexl:** Resources; validation. **Katrien de Bock:** Resources; methodology. **Christian Stockmann:** Conceptualization; resources; data curation; supervision; funding acquisition; methodology; writing – original draft; project administration; writing – review and editing.

Disclosure and competing interests statement

The authors declare that they have no conflict of interest.

References

Assmann N, O'Brien KL, Donnelly RP, Dyck L, Zaiatz-Bittencourt V, Loftus RM, Heinrich P, Oefner PJ, Lynch L, Gardiner CM *et al* (2017) Srebp-controlled glucose metabolism is essential for NK cell functional responses. *Nat Immunol* 18: 1197 – 1206

Crabtree HG (1929) Observations on the carbohydrate metabolism of tumours. *Biochem J* 23: 536 – 545

Donnelly RP, Loftus RM, Keating SE, Liou KT, Biron CA, Gardiner CM, Finlay DK (2014) mTORC1-dependent metabolic reprogramming is a prerequisite for NK cell effector function. *J Immunol* 193: 4477 – 4484

Fang EF, Kassahun H, Croteau DL, Scheibye-Knudsen M, Marosi K, Lu H, Shamanna RA, Kalyanasundaram S, Bollineni RC, Wilson MA *et al* (2016) NAD+ replenishment improves lifespan and Healthspan in ataxia telangiectasia models via mitophagy and DNA repair. *Cell Metab* 24: 566 – 581

Gardiner CM, Finlay DK (2017) What fuels natural killers? Metabolism and NK cell responses. *Front Immunol* 8: 367

Hayakawa Y, Smyth MJ (2006) CD27 dissects mature NK cells into two subsets with distinct responsiveness and migratory capacity. *J Immunol* 176: 1517 – 1524

Huntington ND, Vosshenrich CAJ, di Santo JP (2007) Developmental pathways that generate natural-killer-cell diversity in mice and humans. *Nat Rev Immunol* 7: 703 – 714

Isaacson B, Mandelboim O (2018) Sweet killers: NK cells need glycolysis to kill tumors. *Cell Metab* 28: 183 – 184

Iyer N v, Kotch LE, Agani F, Leung SW, Laughner E, Wenger RH, Gassmann M, Gearhart JD, Lawler AM, Yu AY *et al* (1998) Cellular and developmental control of O_2 homeostasis by hypoxia- inducible factor 1α. *Genes Dev* 12: 149 – 162

Jagannathan L, Cuddapah S, Costa M (2016) Oxidative stress under ambient and physiological oxygen tension in tissue culture. *Curr Pharmacol Rep* 2: 64 – 72

Kaelin WG (2008) The von Hippel–Lindau tumour suppressor protein: O_2 sensing and cancer. *Nat Rev Cancer* 8: 865 – 873

Keppel MP, Saucier N, Mah AY, Vogel TP, Cooper MA (2015) Activation-specific metabolic requirements for NK cell IFN-γ production. *J Immunol* 194: 1954 – 1962

Kim JW, Tchernyshyov I, Semenza GL, Dang CV (2006) HIF-1-mediated expression of pyruvate dehydrogenase kinase: a metabolic switch required for cellular adaptation to hypoxia. *Cell Metab* 3: 177 – 185

Krzywinska E, Kantari-Mimoun C, Kerdiles Y, Sobecki M, Isagawa T, Gotthardt D, Castells M, Haubold J, Millien C, Viel T *et al* (2017) Loss of HIF-1α in natural killer cells inhibits tumour growth by stimulating non-productive angiogenesis. *Nat Commun* 8: 1597

Krzywinska E, Sobecki M, Nagarajan S, Zacharjasz J, Tambuwala MM, Pelletier A, Cummins E, Gotthardt D, Fandrey J, Kerdiles YM *et al* (2022)

The transcription factor HIF-1α mediates plasticity of NKp46+ innate lymphoid cells in the gut. *J Exp Med* 219: e20210909

Li M, Kim WY (2011) Two sides to every story: the HIF-dependent and HIF-independent functions of pVHL. *J Cell Mol Med* 15: 187–195

Loftus RM, Assmann N, Kedia-Mehta N, O'Brien KL, Garcia A, Gillespie C, Hukelmann JL, Oefner PJ, Lamond AI, Gardiner CM *et al* (2018) Amino acid-dependent cMyc expression is essential for NK cell metabolic and functional responses in mice. *Nat Commun* 9: 152–160

Luengo A, Li Z, Gui DY, Sullivan LB, Zagorulya M, Do BT, Ferreira R, Naamati A, Ali A, Lewis CA *et al* (2021) Increased demand for NAD+ relative to ATP drives aerobic glycolysis. *Mol Cell* 81: 691–707

Mace EM, Dongre P, Hsu H-T, Sinha P, James AM, Mann SS, Forbes LR, Watkin LB, Orange JS (2014) Cell biological steps and checkpoints in accessing NK cell cytotoxicity. *Immunol Cell Biol* 92: 245–255

Mah LJ, El-Osta A, Karagiannis TC (2010) γH2AX: a sensitive molecular marker of DNA damage and repair. *Leukemia* 24: 679–686

Marçais A, Cherfils-Vicini J, Viant C, Degouve S, Viel S, Fenis A, Rabilloud J, Mayol K, Tavares A, Bienvenu J *et al* (2014) The metabolic checkpoint kinase mTOR is essential for IL-15 signaling during the development and activation of NK cells. *Nat Immunol* 15: 749–757

Murphy MP (2009) How mitochondria produce reactive oxygen species. *Biochem J* 417: 1–13

Ni J, Wang X, Stojanovic A, Zhang Q, Wincher M, Bühler L, Arnold A, Correia MP, Winkler M, Koch P-S *et al* (2020) Single-cell RNA sequencing of tumor-infiltrating NK cells reveals that inhibition of transcription factor HIF-1α unleashes NK cell activity. *Immunity* 52: 1075–1087

Papandreou I, Cairns RA, Fontana L, Lim AL, Denko NC (2006) HIF-1 mediates adaptation to hypoxia by actively downregulating mitochondrial oxygen consumption. *Cell Metab* 3: 187–197

Poznanski SM, Barra NG, Ashkar AA, Schertzer JD (2018) Immunometabolism of T cells and NK cells: metabolic control of effector and regulatory function. *Inflamm Res* 67: 813–828

Schofield CJ, Ratcliffe PJ (2004) Oxygen sensing by HIF hydroxylases. *Nat Rev Mol Cell Biol* 5: 343–354

Sedlackova L, Korolchuk VI (2020) The crosstalk of NAD, ROS and autophagy in cellular health and ageing. *Biogerontology* 21: 381–397

Semenza GL (2012) Hypoxia-inducible factors in physiology and medicine. *Cell* 148: 399–408

Sobecki M, Krzywinska E, Nagarajan S, Audigé A, Huỳnh K, Zacharjasz J, Debbache J, Kerdiles Y, Gotthardt D, Takeda N *et al* (2021) NK cells in hypoxic skin mediate a trade-off between wound healing and antibacterial defence. *Nat Commun* 12: 1–15

Spits H, Bernink JH, Lanier L (2016) NK cells and type 1 innate lymphoid cells: partners in host defense. *Nat Immunol* 17: 758–764

Trinchieri G (1989) Biology of natural killer cells. *Adv Immunol* 47: 187–376

Ullah MS, Davies AJ, Halestrap AP (2006) The plasma membrane lactate transporter MCT4, but not MCT1, is up-regulated by hypoxia through a HIF-1α-dependent mechanism. *J Biol Chem* 281: 9030–9037

Veys K, Alvarado-Diaz A, de Bock K (2019) Measuring glycolytic and mitochondrial fluxes in endothelial cells using radioactive tracers. *Methods Mol Biol* 1862: 121–136

Vivier E, Nunès JA, Vély F (2004) Natural killer cell signaling pathways. *Science* 306: 1517–1519

Vivier E, Ugolini S, Blaise D, Chabannon C, Brossay L (2012) Targeting natural killer cells and natural killer T cells in cancer. *Nat Rev Immunol* 12: 239–252

Zhang H, Bosch-Marce M, Shimoda LA, Yee ST, Jin HB, Wesley JB, Gonzalez FJ, Semenza GL (2008) Mitochondrial autophagy is an HIF-1-dependent adaptive metabolic response to hypoxia. *J Biol Chem* 283: 10892–10903

License: This is an open access article under the terms of the Creative Commons Attribution License, which permits use, distribution and reproduction in any medium, provided the original work is properly cited.

© 2023 The Authors

Expanded View Figures

Figure EV1. HIF1α is involved in tryptophan/NAD metabolism of splenic NK cells.

A, B NK cell, defined as $CD45^{+}$ $CD3^{-}$ $NKp46^{+}$ $CD49b^{+}$, (A) count and (B) frequency in bone marrow of WT and HIF-1α KO mice ($n = 3$).
C, D NK cell (A) count and (D) frequency in liver of WT and HIF-1α KO mice ($n = 3$).
E Gene expression analysis of genes from the NAD—tryptophan pathway and key amino acid transporters from bulk RNA-sequencing performed on six samples of freshly isolated NK cells from WT and HIF-1α KO mice.
F The tryptophan pathway (Trp, tryptophan; NFK, N-formylkynurenine; Kyn, kynurenine; KA, kynurenic acid; 3-HK, 3-hydroxykynurenine; 3-HAA, 3-hydroxyanthranilic acid; ACMS, 2-amino-3-carboximuconate semialdehyde; AMS, aminomuconate semialdehyde; QA, quinolinic acid) and GSEA analysis of the RNA expression of its key enzymes (Ido, indoleamine-2,3-dioxygenase; Tdo, tryptophan-2,3-dioxygenase; Afmid, kynurenine formamidase; Kyat, kynurenine aminotransferase; Kmo, kynurenine 3-monooxygenase; Kynu, kynureninase; Haao, 3-hydroxyanthranilate 3,4-dioxygenase; Acmsd, ACMS decarboxylase).
G Analysis of quinolinic acid quantification from targeted metabolomics.
H Analysis of mitochondrial ROS amount of freshly isolated NK cells from bone marrow (BM) and liver of WT and HIF-1α KO mice ($n = 3$).
I Analysis of DNA damage in bone marrow (BM) and liver NK cells from WT and HIF-1α KO mice by FACS measurement of γ-H2AX ($n = 3$).

Data information: Statistical significance was determined by an unpaired Student's *t*-test. Bars represent mean values, error bars indicate the s.e.m., (*n*) represents the number of independent experiments, and each data point represents a biological sample from a mouse.
Source data are available online for this figure.

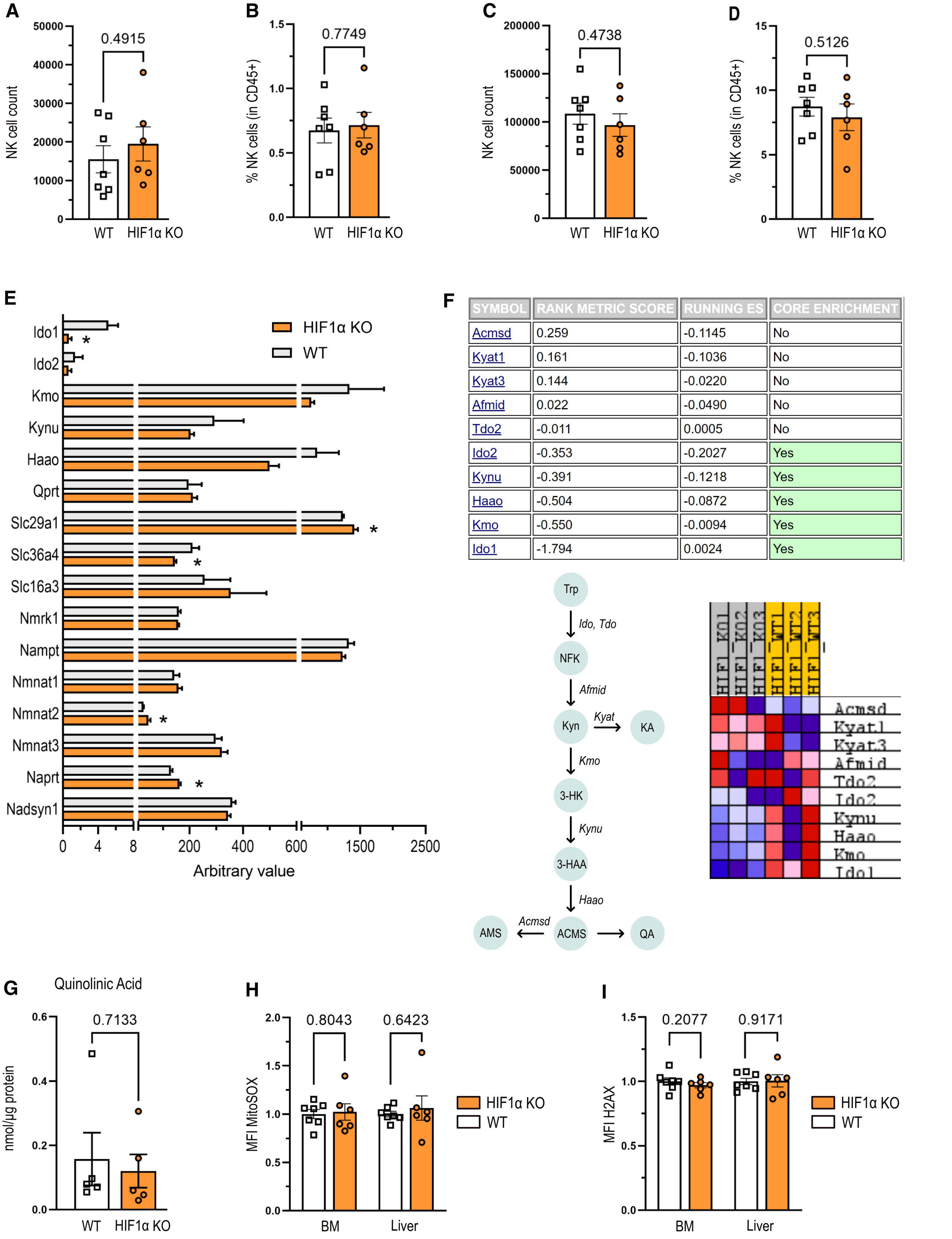

SYMBOL	RANK METRIC SCORE	RUNNING ES	CORE ENRICHMENT
Acmsd	0.259	-0.1145	No
Kyat1	0.161	-0.1036	No
Kyat3	0.144	-0.0220	No
Afmid	0.022	-0.0490	No
Tdo2	-0.011	0.0005	No
Ido2	-0.353	-0.2027	Yes
Kynu	-0.391	-0.1218	Yes
Haao	-0.504	-0.0872	Yes
Kmo	-0.550	-0.0094	Yes
Ido1	-1.794	0.0024	Yes

Figure EV1.

 © 2023 The Authors

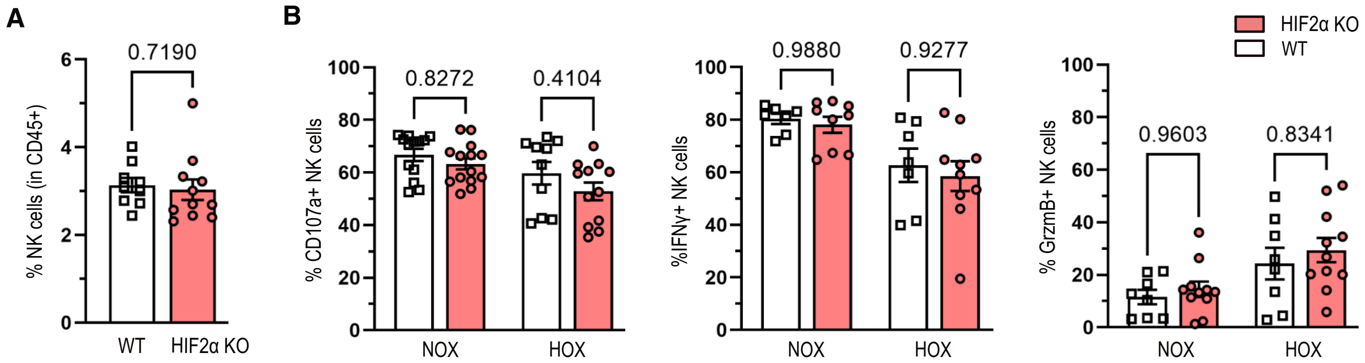

Figure EV2. HIF2α does not play a major role for homeostasis and activation of NK cells.

A NK cell frequency, defined as $CD45^+$ $CD3^-$ $NK1.1^+$ $NKp46^+$, in spleens from WT and HIF-2α KO mice ($n = 3$).

B Splenocytes of WT and HIF-2α KO mice were stimulated with PMA (200 ng/ml) and ionomycin (1 μg/ml) for 6 h at 37°C, 5% CO_2 and 20% O_2 (NOX) or 2% O_2 (HOX) in complete RPMI medium. Degranulation (CD107a) ($n = 3$), IFN-γ ($n = 2$) and granzyme B ($n = 4$) expression were analyzed by flow cytometry.

Data information: Statistical significance was determined by an unpaired Student's *t*-test, one-sample *t*-test or one-way ANOVA where appropriate. Bars represent mean values, error bars indicate the s.e.m., (n) represents the number of independent experiments, and each data point represents a biological sample from a mouse.
Source data are available online for this figure.

© 2023 The Authors
