# Supplementary figures and images for: Resting natural killer cell homeostasis relies on tryptophan/NAD + metabolism and HIF‐1α
Source: EMBO Rep. 2023 Mar 29;24(6):e56156. doi: 10.15252/embr.202256156 (PMC10240188; doi:10.15252/embr.202256156)

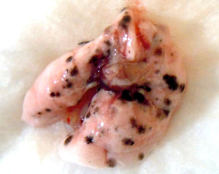

Supplement: Supplementary file 6 — Source Data for Figure 3 [file EMBR-24-e56156-s002.zip › Figure 3/3E_WT.tif]

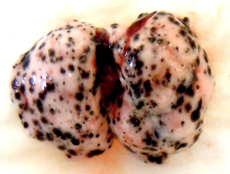

Supplement: Supplementary file 6 — Source Data for Figure 3 [file EMBR-24-e56156-s002.zip › Figure 3/3E_HIF1KO.tif]

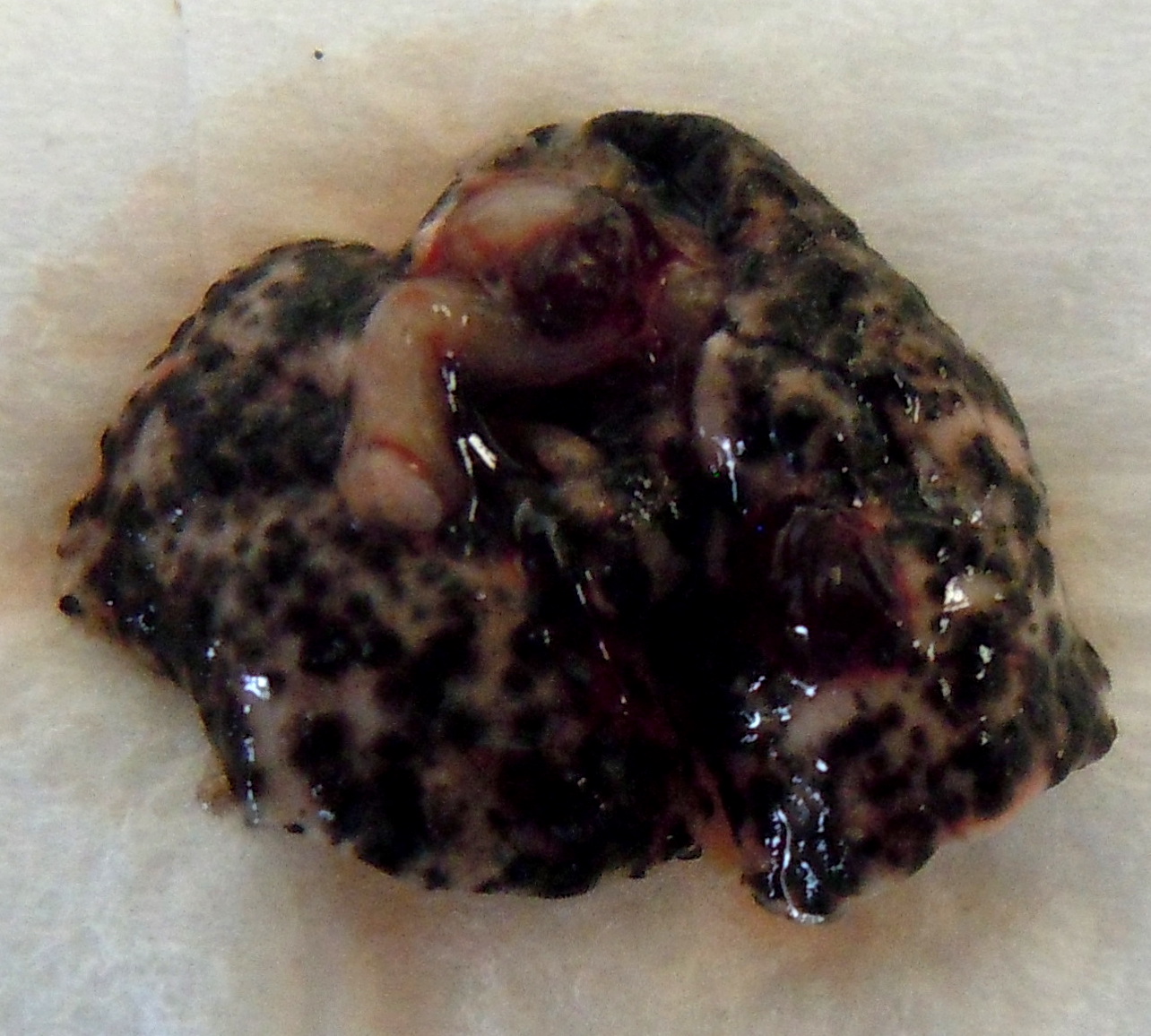

Supplement: Supplementary file 7 — Source Data for Figure 4 [file EMBR-24-e56156-s008.zip › Figure 4/4G_WT.JPG]

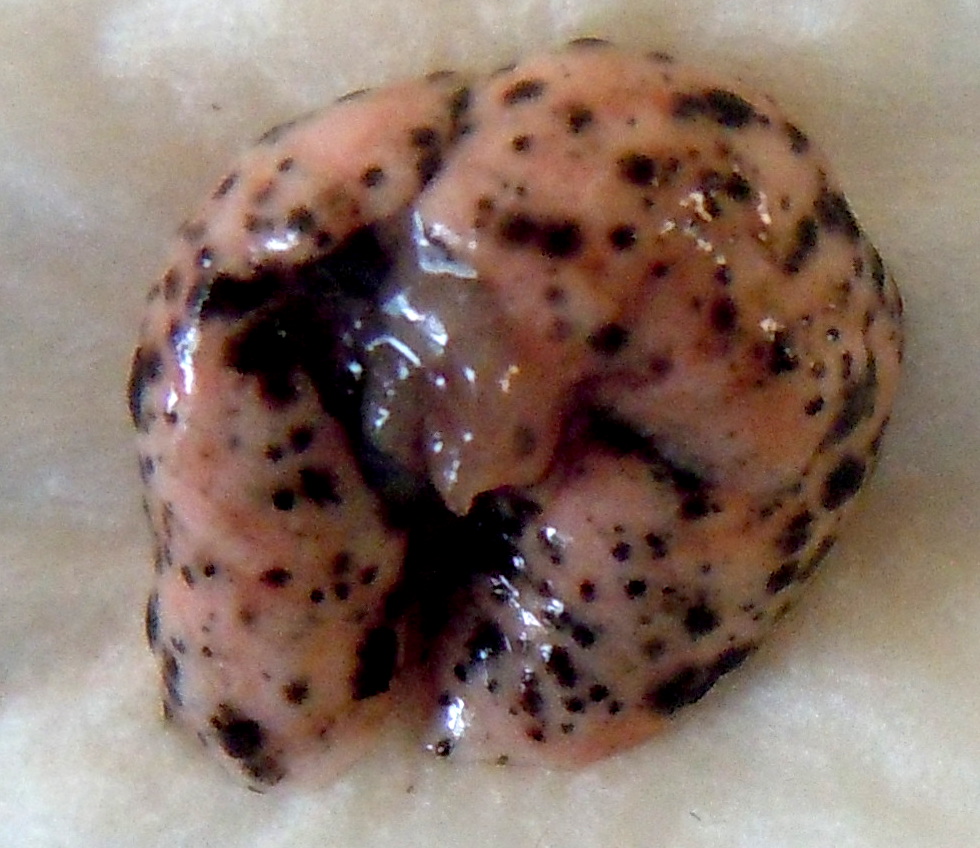

Supplement: Supplementary file 7 — Source Data for Figure 4 [file EMBR-24-e56156-s008.zip › Figure 4/4G_VHL KO.JPG]
