# Supplementary material for: Resting natural killer cell homeostasis relies on tryptophan/NAD + metabolism and HIF‐1α
Source: EMBO Rep. 2023 Mar 29;24(6):e56156. doi: 10.15252/embr.202256156 (PMC10240188; doi:10.15252/embr.202256156)
Supplement: Supplementary file 8 [file EMBR-24-e56156-s003.pdf]

EMBO reports

Resting Natural Killer cell homeostasis relies on tryptophan/NAD+ metabolism and HIF-1α

Abigaelle Pelletier, Eric Nelius, Zheng Fan, Ekaterina Khatchatourova, Abdiel Alvarado-Diaz, Jingyi He, Ewelina Krzywinska, Michal Sobecki, Shunmugam Nagarajan, Yann Kerdiles, Joachim Fandrey, Dagmar Gotthardt, Veronika Sexl, Katrien De Bock, and Christian Stockmann
DOI: 10.15252/embr.202256156

Corresponding author: Christian Stockmann (christian.stockmann@uzh.ch)

Review Timeline:

Submission Date:	20th Sep 22
Editorial Decision:	28th Oct 22
Revision Received:	1st Feb 23
Editorial Decision:	24th Feb 23
Revision Received:	5th Mar 23
Accepted:	10th Mar 23

Editor: Ioannis Papaioannou

Transaction Report:

(Note: With the exception of the correction of typographical or spelling errors that could be a source of ambiguity, letters and reports are not edited. Depending on transfer agreements, referee reports obtained elsewhere may or may not be included in this compilation. Referee reports are anonymous unless the Referee chooses to sign their reports.)

Dear Dr. Stockmann,

Thank you for the submission of your manuscript to EMBO reports. It has now been seen by three experts in the field, and their detailed reports are appended below.

As you will see, the referees provided broadly favorable and supportive reports. They all acknowledge that the study is well performed and that the findings are novel and interesting. However, they also identify some limitations and provide a number of suggestions for further improvement of the study and the manuscript.

Given these constructive comments, we would like to invite you to revise your manuscript with the understanding that the referee concerns (as detailed in their reports) must be fully addressed and their suggestions taken on board. Please address all referee concerns in a complete point-by-point response. Acceptance of the manuscript will depend on a positive outcome of a second round of review. It is EMBO reports policy to allow a single round of revision only and acceptance or rejection of the manuscript will therefore depend on the completeness of your responses included in the next, final version of the manuscript. If you have any questions or comments, we can also discuss the revisions in a video chat, if you like.

We realize that it is difficult to revise to a specific deadline. In the interest of protecting the conceptual advance provided by the work, we usually recommend a revision within 3 months (January 27th). Please discuss with me the revision progress ahead of this time if you require more time to complete the revisions.

Please note that you can publish the study either as a short report or as a full article. For short reports, the revised manuscript should not exceed 27,000 characters (including spaces but excluding materials & methods and references) and 5 main plus 5 expanded view figures. The results and discussion sections must further be combined, which will help to shorten the manuscript text by eliminating some redundancy that is inevitable when discussing the same experiments twice. For a normal article there are no length limitations, but it should have more than 5 main figures and the results and discussion sections must be separate. In both cases, the entire materials and methods must be included in the main manuscript file.

*****
IMPORTANT NOTE:
We perform an initial quality control of all revised manuscripts before re-review. Your manuscript will FAIL this control and the handling will be DELAYED if the following APPLIES:

1) A data availability section providing access to data deposited in public databases is missing.

2) Your manuscript contains statistics and error bars based on n=2. Please use scatter plots in these cases. No statistics should be calculated if n=2.

When submitting your revised manuscript, please carefully review the instructions that follow below. Failure to include requested items will delay the evaluation of your revision.
*****

When submitting your revised manuscript, we will require:

1) a .docx formatted version of the manuscript text (including legends for main figures, EV figures and tables). Please make sure that the changes are highlighted to be clearly visible.

2) individual production quality figure files as .eps, .tif, .jpg (one file per figure).
Please download our Figure Preparation Guidelines (figure preparation pdf) from our Author Guidelines pages https://www.embopress.org/page/journal/14693178/authorguide for more info on how to prepare your figures.

3) a .docx formatted letter INCLUDING the reviewers' reports and your detailed point-by-point responses to their comments. As part of the EMBO Press transparent editorial process, the point-by-point response is part of the Review Process File (RPF), which will be published alongside your paper unless you opt out of this (please see below for further information).

4) a complete author checklist, which you can download from our author guidelines (<https://www.embopress.org/page/journal/14693178/authorguide>). Please insert information in the checklist that is also reflected in the manuscript. The completed author checklist will also be part of the RPF.

5) Please note that all corresponding authors are required to supply an ORCID ID for their name upon submission of a revised manuscript (<https://orcid.org/>). Please find instructions on how to link your ORCID ID to your account in our manuscript

tracking system in our Author guidelines
(<https://www.embopress.org/page/journal/14693178/authorguide#authorshipguidelines>)

6) We replaced Supplementary Information with Expanded View (EV) Figures and Tables that are collapsible/expandable online. A maximum of 5 EV Figures can be typeset. EV Figures should be cited as 'Figure EV1, Figure EV2" etc... in the text and their respective legends should be included in the main text after the legends of regular figures.

- For the figures that you do NOT wish to display as Expanded View figures, they should be bundled together with their legends in a single PDF file called *Appendix*, which should start with a short Table of Content. Appendix figures should be referred to in the main text as: "Appendix Figure S1, Appendix Figure S2" etc. See detailed instructions regarding expanded view here:
<https://www.embopress.org/page/journal/14693178/authorguide#expandedview>

- Additional Tables/Datasets should be labeled and referred to as Table EV1, Dataset EV1, etc. Legends have to be provided in a separate tab in case of .xls files. Alternatively, the legend can be supplied as a separate text file (README) and zipped together with the Table/Dataset file.

7) Before submitting your revision, primary datasets (and computer code, where appropriate) produced in this study need to be deposited in an appropriate public database (see <
https://www.embopress.org/page/journal/14693178/authorguide#dataavailability>).
Specifically, we would kindly ask you to provide public access to the following datasets/data:
- Metabolomic data
- RNA sequencing data

Please remember to provide a reviewer password if the datasets are not yet public.

The accession numbers and database should be listed in a formal "Data Availability " section (placed after Materials & Methods) that follows the model below (see also < https://www.embopress.org/page/journal/14693178/authorguide#dataavailability>). Please note that the Data Availability Section is restricted to new primary data that are part of this study.

Data availability

The datasets (and computer code) produced in this study are available in the following databases:

- RNA-Seq data: Gene Expression Omnibus GSE46843 (https://www.ncbi.nlm.nih.gov/geo/query/acc.cgi?acc=GSE46843)
- [data type]: [name of the resource] [accession number/identifier/doi] ([URL or identifiers.org/DATABASE:ACCESSION])

*** Note - All links should resolve to a page where the data can be accessed. ***

8) We updated our journal's competing interests policy in January 2022 and request authors to consider both actual and perceived competing interests. Please review the new policy (<https://www.embopress.org/competing-interests>) and update your competing interests statement if necessary. Please name this section 'Disclosure and competing interests statement' and place it after the Acknowledgements section.

9) Figure legends and data quantification:
The following points must be specified in each figure legend:

- the name of the statistical test used to generate error bars and P values,
- the number (n) of independent experiments (please specify technical or biological replicates) underlying each data point,
- the nature of the bars and error bars (s.d., s.e.m.)
- If the data are obtained from n {less than or equal to} 2, use scatter plots showing the individual data points.

Discussion of statistical methodology can be reported in the materials and methods section, but figure legends should contain a basic description of n, P and the test applied.

See also the guidelines for figure legend preparation:
https://www.embopress.org/page/journal/14693178/authorguide#figureformat

- Please also include scale bars in all microscopy images.

10) At EMBO Press we ask authors to provide source data for the main and EV figures. Our source data coordinator will contact you to discuss which figure panels we would need source data for and will also provide you with helpful tips on how to upload and organize the files.

11) Our journal encourages inclusion of *data citations in the reference list* to directly cite datasets that were re-used and

obtained from public databases. Data citations in the article text are distinct from normal bibliographical citations and should directly link to the database records from which the data can be accessed. In the main text, data citations are formatted as follows: "Data ref: Smith et al, 2001" or "Data ref: NCBI Sequence Read Archive PRJNA342805, 2017". In the Reference list, data citations must be labeled with "[DATASET]". A data reference must provide the database name, accession number/identifiers and a resolvable link to the landing page from which the data can be accessed at the end of the reference. Further instructions are available at <https://www.embopress.org/page/journal/14693178/authorguide#referencesformat>.

12) Please also note our reference format:
<http://www.embopress.org/page/journal/14693178/authorguide#referencesformat>.

13) We now use CRediT to specify the contributions of each author in the journal submission system. CRediT replaces the author contribution section, which should be removed from the manuscript. Please use the free text box to provide more detailed descriptions. See also guide to authors:
<https://www.embopress.org/page/journal/14693178/authorguide#authorshipguidelines>.

14) As part of the EMBO publications' Transparent Editorial Process, EMBO reports publishes online a Review Process File to accompany accepted manuscripts. This File will be published in conjunction with your paper and will include the referee reports, your point-by-point response and all pertinent correspondence relating to the manuscript.

You can opt out of this by letting the editorial office know (emboreports@embo.org). If you do opt out, the Review Process File link will point to the following statement: "No Review Process File is available with this article, as the authors have chosen not to make the review process public in this case."

We would also welcome the submission of cover suggestions, or motifs to be used by our Graphics Illustrator in designing a cover.

I look forward to seeing a revised version of your manuscript when it is ready. Please let me know if you have questions or comments regarding the revision.

Kind regards,

Ioannis

Ioannis Papaioannou, PhD
Editor
EMBO reports

Referee #1:

In this study Pelletier et al. investigate the role of HIF1a in NK cell homeostasis under hypoxic conditions. Using mouse models of NK cell specific HIF1a KO and constitutive HIF1a activation, they show that NK cell homeostasis under steady state conditions is regulated by HIF1a dependent tryptophan/NAD metabolism while NK cell activation is dependent on HIF1a mediated glycolysis. Overall, the study is well performed, the data is well presented, and the findings add new insights into the role of HIF1a in NK cells under hypoxic conditions. The topic of the study is of broad interest since NK cells are important effector cells in anti-microbial and anti-tumor immunity that often have to exert their functions in hypoxic tissue environments. However, I have a few concerns/questions that the authors should address:

1. To better understand the role of HIF1a in NK cells under hypoxic homeostatic conditions in vivo it would be informative to perform some analysis not only on splenic NK cells but also on NK cells isolated from other organs with higher oxygen pressures, such as liver and bone marrow (as mentioned by the authors on page 4). Do HIF1a KO mice only exhibit reduced numbers of NK cells (Fig.1a) in the spleen (due to its low oxygen level) while NK cell numbers in organs with higher oxygen levels are comparable to WT mice? In addition, do splenic NK cells show higher expression levels of HIF1a (protein or mRNA) as compared to NK cells from liver or bone marrow in WT mice? Finally, are the differences between WT and KO mice observed in the metabolomic and transcriptomic analysis (Fig.1c-d) only detectable in splenic NK cells but not in NK cells directly isolated from non-hypoxic tissue environments?
2. In Fig.2 j-k it seems that NK cells from WT mice (just focusing on the white bars) show significantly higher IFN-g production

under HOX conditions (with or without NAD or NAM) as compared to NOX conditions. How can that be explained?
3. In Fig. 3a-c please show total cell numbers of these cell subsets in addition to percentages.
4. The results from the tumor model (Fig.3 i-j) are not consistent with results previously published by the authors (Krzywinska et al. 2017) and other groups (Ni et al. 2020). In these studies mice with NK specific HIF1a KO showed reduced tumor burden as compared to controls. While these divergent results can probably be explained by the different tumor models used, the authors should discuss their current results in the context of these previous findings. In addition, please describe the tumor model in more detail in the material and methods section as there is currently very little information available on how these experiments were performed. Only a very brief description is provided in the caption of Fig.3. Why is the tumor burden in WT mice in Fig.3i much lower than the tumor burden in WT mice in Fig. 3j? Was a different model used in these two experiments?
5. Related to point 4, in Figure 3 please provide data on the effector functions (CD107a, IFN-g) of tumor-infiltrating NK cells in WT, HIF1a KO and VHL KO mice to confirm that differences in tumor burden are in fact due to differences in NK cell function in these different mouse strains.
6. Please provide more information about the experimental mice in the material and methods section. What genetic background? Age of mice at time of experiments? Were male and female mice used at equal numbers?

Referee #2:

Pelletier et al. describe the metabolic, phenotypic and functional responses of HIF1a knock-out NK cells freshly isolated from healthy mice. A detailed metabolic screen revealed unexpected depletion of tryptophan and dysregulation of the NAD pathway, alongside more abundant reactive oxygen species, DNA damage and apoptosis. They also observed subtle defects in glycolysis, degranulation and cytokine production in the absence of HIF1a. All these compound into a very striking defect in tumor control in a B16F10 model. The authors then examined how knocking out tumor suppressor gene, Von Hippel Lindau, in NK cells, which targets HIF1a for degradation (among other proteins), resulted in increases in glycolytic function, granzyme B content and tumor control. Overall the data are interesting and the experiments are carefully and thoughtfully produced. The work interesting and robust, but the authors more clearly state the limitations of the study. Specifically:
1. The authors should mention that VHL has been reported to target atypical protein kinase C and RNA polymerase II for ubiquitination. HIF1a is the most likely target, given the HIF2a control described by the authors. Nevertheless, it is worth noting that other (less likely) explanations are possible.
2. The authors have described defects in degranulation in response to non-specific stimuli (PMA/ionomycin) but have not shown impaired cytotoxicity of tumor cells directly. It is possible that cytotoxicity is more profoundly affected or is unaffected, which would also be interesting! If cytotoxicity is unaffected, it might suggest infiltration into the hypoxic tumor microenvironment might be part of the mechanism here.
3. The histological features of the tumor and lack or not of NK cell penetrance into the tumor has not been addressed here either. NK cell infiltration studies would be an interesting further dissection of this model. At the very least, these limitations of the study should be acknowledged.
4. Some comment should be made on how the authors believe such a robust effect is seen in vivo, from remarkably subtle changes in apoptosis and degranulation.
5. One of the most interesting points of this study is that it conflicts with the report by Ni et al. Immunity 2020 (10.1016/j.immuni.2020.05.001). The authors should discuss why they think their studies differ from those of Ni et al. to give a perspective on the differences.
6. There are some important omissions in the methods section. What are the details of the B16F10 mouse model? How many tumor cells were injected per animal? How was the granzyme B measured following PMA/ionomycin stimulation performed? Is this intracellular or secreted granzyme B? Providing these details would allow a more in depth discussion of how this manuscript differs from the models put forward by Ni et al.
7. There are also a number of typos in the document that should be corrected e.g. 'cytoxic' rather than 'cytotoxic' in the introduction.
8. I believe Figure 2i is mislabeled in the legend.

Referee #3:

The report by Pelletier et al. describes the in vivo consequences of HIF loss-of-function or gain-of-function mutations in resting and activated NK cells using conditional knockout mice. In addition to providing evidence in support of a role of HIF-1 in activated NK cells residing in a hypoxic environment, the authors make a novel link of HIF-1 signaling with tryptophan and nicotinamide metabolism in resting NK cells, which has functional consequences on OxPhos-associated mitochondrial ROS production, DNA damage, and apoptosis. The data include global untargeted metabolomic and transcriptomic measurements of rapidly purified, resting NK cell-enriched cell populations from spleens, which revealed a decrease in tryptophan and NAM levels as well as in mRNA levels for cellular proteins involved in tryptophan uptake and catabolism, respectively. Targeted metabolomics revealed reduced tryptophan catabolites and NAD precursors as well as a decreased free NAD+/NADH ratio. The

authors also report oxygen consumption rate (OCR), fatty acid oxidation, and glutaminolysis data for splenic cells isolated from WT or HIF-1a NK-cell conditional KO mice and perform a subset of these studies with NK cells isolated from NK-cell conditional VHL knockout mice.

A major strength of this study is a focus on the in vivo aspect of NK biology using genetically modified mice, which is the most physiologically relevant model for these studies. The authors use floxed HIF-1a and HIF-2a mice as well as floxed VHL conditional mice in conjunction with an NK cell-restricted Ncr1-Cre driver transgene to examine the effect of impaired or augmented HIF signaling, respectively, in splenic NK cells. The studies are generally robust and the conclusions reasonable. However, there are areas in which the current study could be strengthened. My concerns for this study are as follows.

Figure 1:
• The differences in gene expression data in Figure 1d are a bit difficult to appreciate plotted only in this manner. It may be helpful to add another panel where you normalize each gene to 100% for the WT level and compare the KO in this manner (relative gene expression patterns).
• Moreover, the criteria for "statistical significance" with P>0.05 may be too stringent for this small sample size. I suspect that Kmo and Kynu levels may reach a significance of P>0.10, which would be further supported by the targeted metabolomic data in Fig 1e.
• It appears that Haao levels may have reached the P>0.05 threshold. If so, this should be so designated and the implications commented on.
• It would be helpful to know what gene expression levels are for Qprt, the enzyme catalyzing conversion of quinolinic acid (QA) to NAD.
• In view of the above, it appears that there may be a pathway decrease in synthetic enzymes involved in QA production, consistent with how HIF-1 regulates glycolytic enzymes. This may have important implications outside of this study. A pathway-type analysis should be performed to test this hypothesis (see below for Figure 2h).
• For Figure 1e, if this information is available, it would be helpful to know levels of other metabolites in this pathway besides KYN that are measurable by targeted metabolomics (3-HK, 3-HAA, QA). This may be of particular importance to the oxidative stress assessment since several of these metabolites may have pro- or anti-oxidant effects in other settings.
• To assist scientists outside this field, designations for all genes and proteins in Figure 1e should be clarified in the legend.
• It is important to acknowledge that the NAD/NADH ratio reported in Figure 1f is based upon total NAD and NADH levels, whereas it is free NAD+ and NADH that has regulatory roles. This is a limitation that is frequently not acknowledged.
Figure 2:
• In line with additional measurements of gene expression detailed above for Figure 1d, a GSEA would be helpful for the tryptophan/quinolinic acid biosynthetic pathway outlined in Figure 1e.
• For improved clarity, may be best to state the legend for Figure 2k as "Same as (i) compared to supplementation with 10 mM NAD+ or NAM (n=2)."
Figure 3:
• To confirm the extent of Cre-mediated excision, immunoblots of VHL and HIF-2, (and HIF-1 in Figure 1), protein levels in purified NK cells isolated from HIF-1, HIF-2, or VHL mice and maintained under normoxic or hypoxic conditions should be performed.
• The above is particularly important as the floxed HIF-2 allele following Cre excision reportedly generates a HIF-2 protein that lacks the bHLH domain, but retains the rest of the HIF-2 protein. Thus, this floxed HIF-2 mouse may not generate a true null allele following Cre-mediated excision and instead may be a potential hypomorph allele, which may or may not be deficient in HIF-2 mediated effects. If this floxed HIF-2 allele generates a HIF-2 protein that is detectable by a polyclonal antibody recognizing the carboxy terminus, this should be acknowledged and the above limitations stated.
• The investigators conclude from the data with HIF-2 floxed mice in Figure 3h that Cre-mediated deletion of the floxed exon does not affect the FACS profiling defined by CD107a, IFN-g, or GrzmB detection. However, the sample size for some of the experiments, particularly the hypoxic ones, was much smaller than that defined by CD107a and IFN-g profiling for HIF-1 floxed mice in Figure 2j, and as such may not detect a true difference if there is a smaller effect size. Accordingly, the p-value for the comparisons in Figure 3h should be presented in the figure legend to allow the reader to judge whether this result is significant or not, rather than just deemed non-significant. This approach would be best for all data presented in this study.
• GrzmB profiling was not shown for the NK cells from HIF-1 floxed mice. If performed, it should be shown even if similar as data generated from WT mice.
• Although the VHL null results are highly suggestive, it is unclear why more was not done to clarify the respective in vivo roles for HIF-1 versus HIF-2 using this model. Specifically, use of a VHL/HIF-1 and VHL/HIF-2 doubly floxed mice with the Ncr1-Cre driver would test the contributions of HIF-1 versus HIF-2, respectively, in the VHL knockout phenotype. I recognize that this may be the basis for future experiments.

Referee #1:

In this study Pelletier et al. investigate the role of HIF1a in NK cell homeostasis under hypoxic conditions. Using mouse models of NK cell specific HIF1a KO and constitutive HIF1a activation, they show that NK cell homeostasis under steady state conditions is regulated by HIF1a dependent tryptophan/NAD metabolism while NK cell activation is dependent on HIF1a mediated glycolysis. Overall, the study is well performed, the data is well presented, and the findings add new insights into the role of HIF1a in NK cells under hypoxic conditions. The topic of the study is of broad interest since NK cells are important effector cells in anti-microbial and anti-tumor immunity that often have to exert their functions in hypoxic tissue environments.

OUR REPLY:

We appreciate the overall positive reception of our work.

However, I have a few concerns/questions that the authors should address:
1. To better understand the role of HIF1a in NK cells under hypoxic homeostatic conditions in vivo it would be informative to perform some analysis not only on splenic NK cells but also on NK cells isolated from other organs with higher oxygen pressures, such as liver and bone marrow (as mentioned by the authors on page 4).
Do HIF1a KO mice only exhibit reduced numbers of NK cells (Fig.1a) in the spleen (due to its low oxygen level) while NK cell numbers in organs with higher oxygen levels are comparable to WT mice?

OUR REPLY:

We very much appreciate this comment. We analyzed the frequencies and absolute numbers of NK cells in the bone marrow (BM) and the liver from WT and HIF1a KO mice. As shown below the frequency and number of NK cells in the bone marrow and liver are similar between WT and HIF1a KO mice:

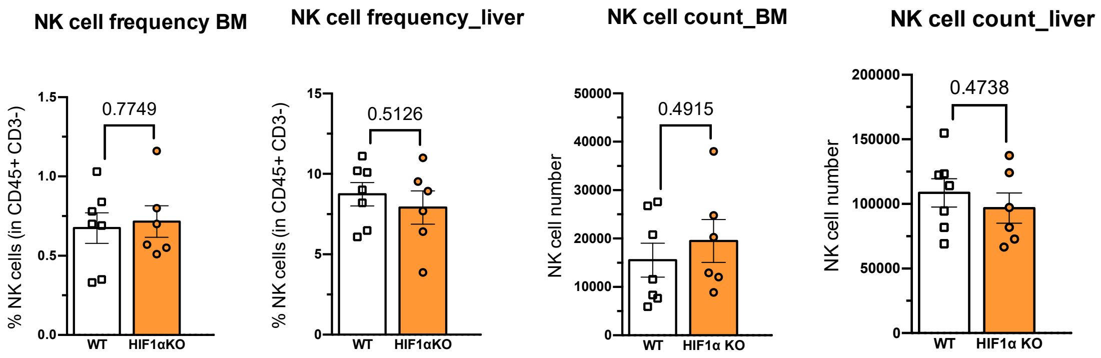

This is also in line with a previous publication from our group (Krzywinska et al. 2017; PMID: 29150606; Suppl. Figure 1a), showing that the abundance of BM and liver NK cells are not affected by loss of HIF1a. The results are now included in the new Suppl. Figure 1 of the revised manuscript and described as follows:

In vivo, targeted deletion of HIF-1α was achieved by crosses of the loxP-flanked HIF-1α allele[17] to the *Ncr1* (NKp46) promoter-driven Cre recombinase[17], specific to NKp46-expressing innate lymphoid cells, including NK cells (HIF-1α$^{fl+/fl+}$/Ncr1^{cre+} mice, termed HIF-1α KO). We first analyzed NK cells in organs, that harbor a substantial number of NK cells but exhibit different oxygen partial pressures (pO_2), namely, the bone marrow (pO_2= 40-50 mmHg), liver (pO_2 = 30-40 mmHg) and spleen (pO_2 = 20 mmHg) (Jagannathan et al., 2016). Mice with an NK cell-specific deletion of HIF-1α, show reduced NK cell numbers in the spleen, hence, the organ with the lowest pO_2 (Fig. 1a), whereas NK cells from the bone marrow and liver were not affected (Suppl. Fig. 1a, b, c and d).

In addition, do splenic NK cells show higher expression levels of HIF1a (protein or mRNA) as compared to NK cells from liver or bone marrow in WT mice?

OUR REPLY:

The reviewer raises an important point. According to the ImmGen database (https://www.immgen.org/Databrowser19/DatabrowserPage.html), bone marrow NK cells have slightly higher HIF1a mRNA expression than splenic NK cells, whereas data on liver NK cells is not deposited in the ImmGen database. We have addressed this further by isolating mRNA from spleen, liver and bone marrow NK cells followed by RT-qPCR for *Hif1a*. As shown below, NK cells from the spleen and bone marrow show similar HIF1a mRNA expression, whereas liver NK cells show increased levels of HIF1a transcript:

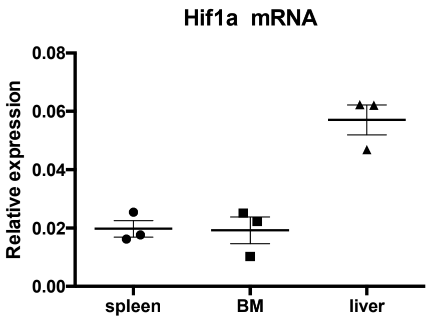

We would like to leave it to the reviewer and the editor whether these data should be included in the main manuscript.

Unfortunately, the assessment and comparison of HIF1a protein levels in NK cells from different organs was not feasible. The time from removing the respective organ and isolating the NK cells to the protein lysate will be at least 60 minutes, yet at 20% oxygen. So, by the time we could eventually get a proteomic snapshot of sorted NK cells, HIF1a protein will be entirely degraded regardless of the genotype.

Finally, are the differences between WT and KO mice observed in the metabolomic and transcriptomic analysis (Fig.1c-d) only detectable in splenic NK cells but not in NK cells directly isolated from non-hypoxic tissue environments?

<u>OUR REPLY:</u>

As outlined above, the absence of HIF1a does not affect NK cell numbers in the bone marrow or liver. In line with this, bone marrow and liver NK cells from HIF1a KO mice do not show elevated ROS levels or increased DNA damage (see below):

f

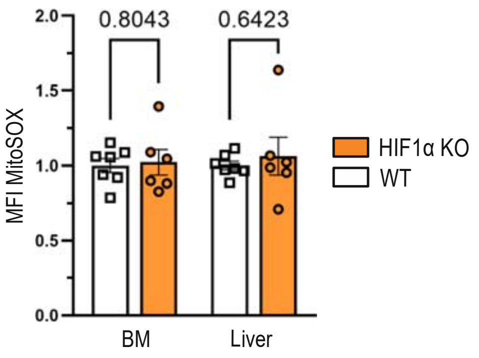

g

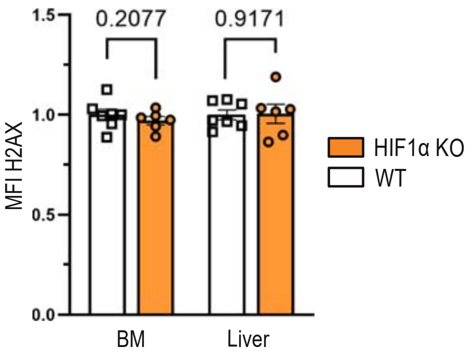

The results are now included in the new Suppl. Figure 1 of the revised manuscript and described as follows:

NAD^+ plays a crucial role in mitochondrial redox homeostasis and DNA damage repair upon oxidative stress (Fang et al., 2016; Sedlackova and Korolchuk, 2020). Consistently, HIF-1α-deficient splenic NK cells generate increased mitochondrial reactive oxidative species (ROS), and suffer from increased DNA damage (Mah et al., 2010) and cell death (Fig. 2a-c). Of note, bone marrow and liver NK cells from HIF1a KO mice do not show elevated ROS levels or increased DNA damage (Suppl. Fig. 1f and g).

Next, we analyzed the mRNA expression of the enzymes of the tryptophan/NAD pathway that are differentially regulated in the RNAseq of splenic HIF1a KO NK cells (*Ido1*, *Slc29a1*, *Slc36a4*, *Nmnat2 and Naprt*) in isolated liver and bone marrow NK cells by RT-qPCR. As shown below *Ido1*, *Slc29a1*, *Slc36a4*, *Nmnat2 and Naprt* show similar expression in WT and HIF1a KO NK cells from the bone marrow and liver:

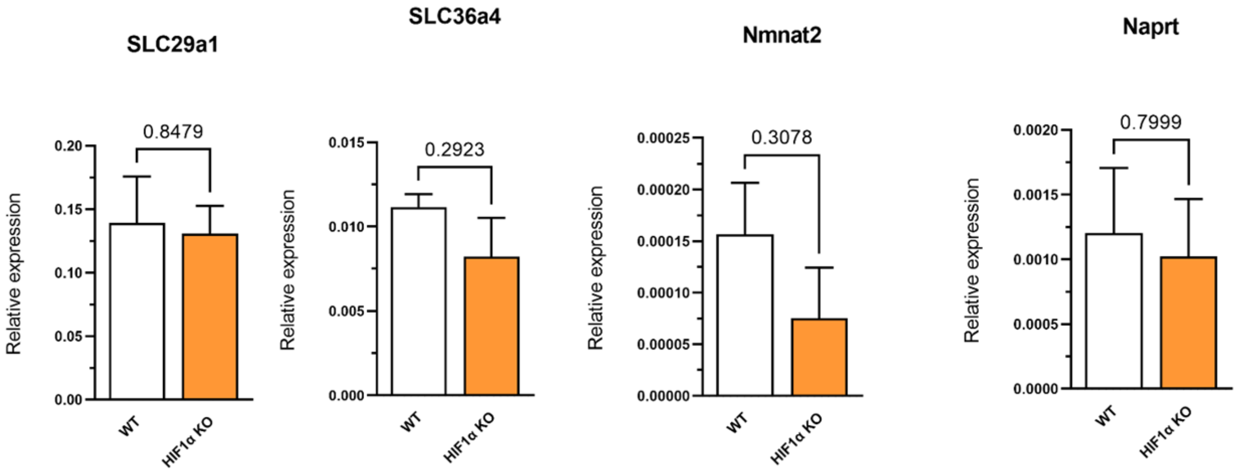

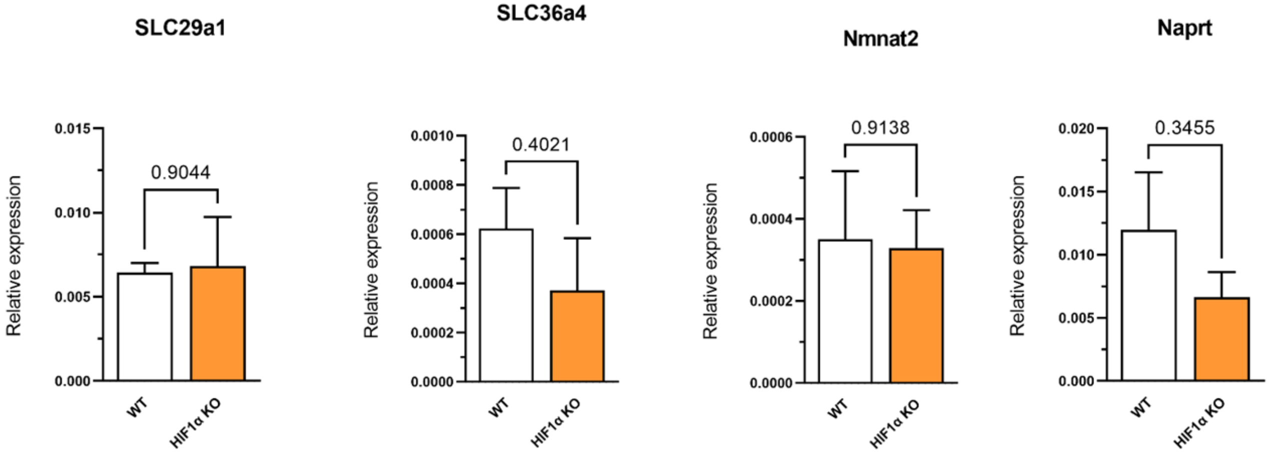

Taken together, this suggests that in contrast to splenic NK cells, HIF1a in bone marrow and liver NK cells is dispensable for homeostasis and maintenance. Therefore, we refrained from repeating the resource-intensive and time-consuming untargeted and targeted metabolomics on NK cells from the liver and the bone marrow.

2. In Fig.2 j-k it seems that NK cells from WT mice (just focusing on the white bars) show significantly higher IFN-g production under HOX conditions (with or without NAD or NAM) as compared to NOX conditions. How can that be explained?

OUR REPLY:

We appreciate this remark and would like to point out that in IFN-g production between WT NK cells in normoxia and hypoxia is indeed different which is now indicated in the graph of the revised Figure 3c. However, given the defect in IFN-g production by HIF1a-deficient cells in normoxia and hypoxia, it is conceivable that hypoxia-driven HIF induction can synergize with activation-driven IFN-g production. It is nevertheless important to emphasize that PMA/ionomycin is a rather strong stimulation and we have previously shown that hypoxia can have differentially impact activation depending on the type of stimulus (Krzywinska et al. 2017; PMID: 29150606; Fig. 1 and Suppl. Figure 1). For instance, hypoxia has the tendency to slightly increase IFN-g secretion upon target cell exposure and stimulation with IL-2 or IL-15. In contrast, IFN-g secretion after stimulation with anti NK1.1 or IL12/18 is not affected by hypoxia (Krzywinska et al. 2017; PMID: 29150606; Fig. 1 and Suppl. Figure 1 therein).

3. In Fig. 3a-c please show total cell numbers of these cell subsets in addition to percentages.

OUR REPLY:

As shown below, we now provide total cell numbers for these subsets:

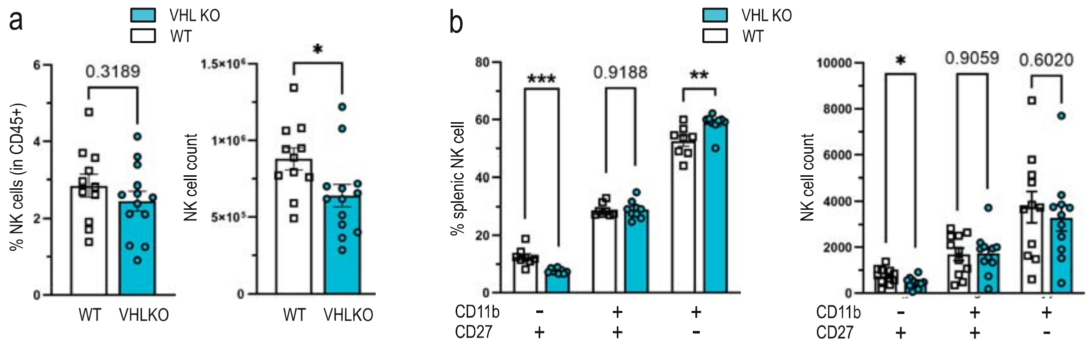

The new results are now included in the new Figure 4 of the revised manuscript as follows:

NK cell reactivity is strongly linked to NK cell maturation, which can be distinguished by the expression of CD27/CD11b [33] along with the development of a repertoire of inhibiting and activating receptors [34]. Importantly, loss of VHL reduced the frequency and number of immature splenic NK cell frequencies (Fig. 4a) but resulted in a slightly increased frequency of mature NK cells in the spleen (Fig. 4b), while the receptor repertoire remained unaffected (Fig. 4c).

4. The results from the tumor model (Fig.3 i-j) are not consistent with results previously published by the authors (Krzywinska et al. 2017) and other groups (Ni et al. 2020). In these studies mice with NK specific HIF1a KO showed reduced tumor burden as compared to controls. While these divergent results can probably be explained by the different tumor models used, the authors should discuss their current results in the context of these previous findings.

OUR REPLY:

We appreciate this comment and would like to point out that all tumor studies in the publication by Krzywinska et al. 2017 were performed with a variety of solid and subcutaneously growing tumors. In this setting, the loss of HIF1a in NK cells leads to excessive formation and remodeling of the tumor vasculature is a major determinant of solid tumor growth as well as intravasation of tumor cells and subsequent

pulmonary metastasis. In the current manuscript, we now injected B16 melanoma cells intravenously in order to circumvent this bias and to exclusively study the impact of HIF1a on NK cell-based immunosurveillance of circulating melanoma cells and pulmonary micrometastasis. In this regard, the results are not necessarily inconsistent as we now show that loss of HIF1a in NK cells leads to impaired control of intravenously injected and circulating melanoma cells. This is at least somewhat consistent with the findings from Krzywinska et al. 2017, where we demonstrate impaired *in vitro* killing (of MHC-I-deficient lymphoma cells) by HIF1a-deficient NK cells.

However, we apologize for the sparse description of the experimental setup to study metastasis, which is now more exhaustively explained in the "methods" section of the revised manuscript as outlined further below in the point-by-point response.

In addition, please describe the tumor model in more detail in the material and methods section as there is currently very little information available on how these experiments were performed. Only a very brief description is provided in the caption of Fig.3. Why is the tumor burden in WT mice in Fig.3i much lower than the tumor burden in WT mice in Fig. 3j? Was a different model used in these two experiments?

OUR REPLY:

We apologize for this oversight. We used indeed a lower number of initially intravenously injected melanoma cells for the HIF1a KO mice in order to anticipate loss of function and compromised NK cell surveillance of melanoma cells along with the associated impact on the well-being of the animals. The "methods" section of the revised manuscript now contains a more detailed description of the procedure as follows:

Pulmonary metastasis model. B16F10 melanoma cell line was culture in a RPMI GlutaMAX TM medium (Thermofisher) supplemented with 10% of fetal bovine serum (FBS) and 50 U/mL penicillin-streptomycin at 37°C and 5% CO_2. Cells were resuspended in PBS and 1×10^5 (for HIF1α mouse model) or 2×10^5 (for VHL mouse model) B16F10 cells in 50 µL of PBS were injected into the tail vein of the animals. The lungs were removed after 7 days for NK

cell infiltration assessment and stimulation assay, and after 14 days for metastatic foci counting.

5. Related to point 4, in Figure 3 please provide data on the effector functions (CD107a, IFN-g) of tumor-infiltrating NK cells in WT, HIF1a KO and VHL KO mice to confirm that differences in tumor burden are in fact due to differences in NK cell function in these different mouse strains.

OUR REPLY:

The reviewer raises a very important point. Hence, given the profound effect of HIF1a on NK cell-dependent pulmonary metastasis in vivo, we decided to analyze abundance and phenotype of NK cells in lungs from mice after i.v. injection of B16 melanoma cells.

As shown below, the increase in pulmonary metastasis HIF1a KO mice is associated with a decrease in pulmonary NK cell frequencies and total numbers, along with a decrease in pulmonary Granzyme B+ and IFNg+ NK cells:

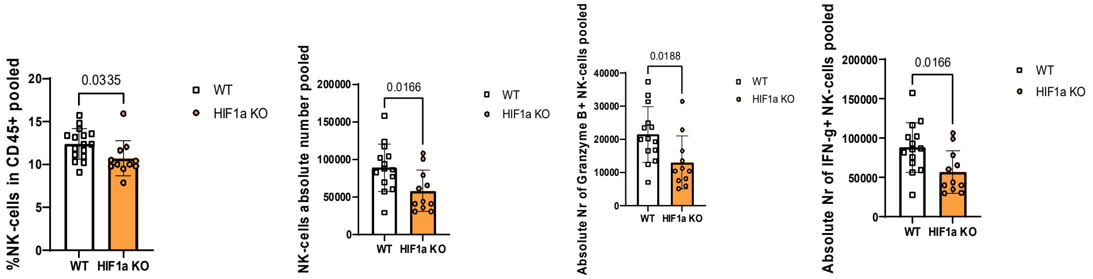

This consistent with previous reports from our lab, where we have shown that loss of HIF1a leads to impaired NK cell infiltration in vivo (Krzywinska et al. 2017).

This data is now included in the new Figure 3 of the revised manuscript and described as follows:

NK cells have a unique capacity to restrict metastatic spread of circulating cancer cells[8]. Therefore, we aimed to determine the impact of HIF-1α expression on NK cell effector function and immunosurveillance of metastatic tumor cells *in vivo*. To this end, we injected syngeneic B16F10 melanoma cells intravenously into HIF-1α KO mice as well as the

corresponding WT littermates and analyzed the number of pulmonary metastases 2 weeks post-injection. Consistent with our *in vitro* observations, loss of HIF-1α in NK cells leads to increased pulmonary metastasis of intravenously injected melanoma cells (Fig. 3i). Given the profound effect of HIF1a on NK cell-dependent pulmonary metastasis in vivo, we decided to analyze abundance and phenotype of NK cells in lungs from mice after i.v. injection of B16 melanoma cells. As shown in Figure 3f, the increase in pulmonary metastasis HIF1a KO mice is associated with a decrease in pulmonary NK cell frequencies and total numbers, along with a decrease in pulmonary Granzyme B+ and IFN-γ + NK cells. This is consistent with previous reports from our lab, where we have shown that loss of HIF1a leads to impaired NK cell infiltration in vivo [20]

Next, we now performed in vitro killing assays with B16 melanoma cells. Spontaneous killing of B16 melanoma cells by NK cells in a standard killing assay (for 6 h at a 10:1 effector/target ratio) without cytokines was only about 2% (data not shown). So, we customized the killing assay for B16 cells by increasing the coincubation to 24 hours and the addition of IL-15 to ensure NK cell survival over this period. However, as shown below, the loss of HIF1a did not affect NK cell-dependent lysis of B16 cells in normoxia or hypoxia (Fig. 3g). This is in line with previous reports from our lab that HIF1a does not affect the in vitro killing of MHC-I-proficient cells [20] and suggests that HIF-1α is required NK cell infiltration into metastatic lesions.

Next, we analyzed the abundance and phenotype of NK cells in lungs from VHL KO mice after i.v. injection of B16 melanoma cells.

As shown below, the decrease in pulmonary metastasis in VHL KO mice is associated with an increase in Granzyme B+ NK cells, whereas the frequency of total NK cells remained unchanged:

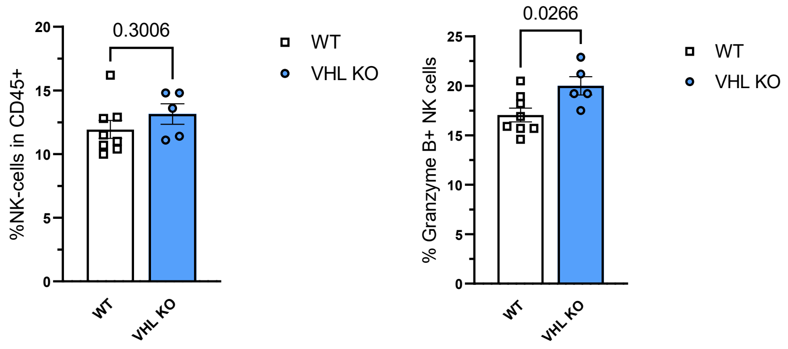

This data is now included in the new Figure 4 of the revised manuscript and described as follows:

Next, we aimed to determine the impact of VHL loss on NK cell immunosurveillance of metastatic tumor cells *in vivo* and injected syngeneic B16F10 melanoma cells intravenously into VHL KO mice as well as the corresponding WT littermates and analyzed the number of pulmonary metastases 2 weeks post-injection. Consistent with our *in vitro* observations, VHL deletion in NK cells reduced pulmonary metastases by almost 70% (Fig. 4g). Furthermore, this was associated with with an increase in Granzyme B+ NK cells, whereas the frequency of total NK cells remained unchanged. (Fig. 4h). This suggests that forced HIF-1α-driven glycolysis can enhance NK cell effector function *in vivo*.

6. Please provide more information about the experimental mice in the material and methods section. What genetic background? Age of mice at time of experiments? Were male and female mice used at equal numbers?

OUR REPLY:

We would like to apologize for this. We used 8-12 weeks old C57Bl/6j mice. Female and mice were used at equal numbers.

This information is now included in the “methods” section of the revised manuscript as follows:

Mouse models. *In vivo*, targeted deletions of HIF-1α and VHL in NK cells were achieved by as previously described (Krzywinska et al., 2017, 2022). To mitigate the influence of strain variation, mice were kept in a > 99% C57Bl/6J background. Male and female mice at the age of 8 to 12 weeks were used at equal ratios.

Referee #2:

Pelletier et al. describe the metabolic, phenotypic and functional responses of HIF1a knock-out NK cells freshly isolated from healthy mice. A detailed metabolic screen revealed unexpected depletion of tryptophan and dysregulation of the NAD pathway, alongside more abundant reactive oxygen species, DNA damage and apoptosis. They also observed subtle defects in glycolysis, degranulation and cytokine production in the absence of HIF1a. All these compound into a very striking defect in tumor control in a B16F10 model. The authors then examined how knocking out tumor suppressor gene, Von Hippel Lindau, in NK cells, which targets HIF1a for degradation (among other proteins), resulted in increases in glycolytic function, granzyme B content and tumor control. Overall the data are interesting and the experiments are carefully and thoughtfully produced. The work interesting and robust, but the authors more clearly state the limitations of the study.

<u>OUR REPLY:</u>

We appreciate the overall positive reception of our work and agree that some of the limitations of our study need be stated more clearly as further outline below.

Specifically:
1. The authors should mention that VHL has been reported to target atypical protein kinase C and RNA polymerase II for ubiquitination. HIF1a is the most likely target, given the HIF2a control described by the authors. Nevertheless, it is worth noting that other (less likely) explanations are possible.

<u>OUR REPLY:</u>

The reviewer raises an important point that we now address by highlighting potential HIF-independent effects of VHL deletion in the combined "results and discussion" section of the manuscript as follows:

As VHL negatively regulates both HIF isoforms, the deletion of VHL not only stabilizes HIF1α but extends to the constitutive expression of HIF-2α. To control for these effects we crossed the loxP-flanked *Epas1* allele[24] that encodes for HIF-2α to the *Ncr1* (NKp46) promoter-driven Cre recombinase[23] ($EPAS1^{fl+/fl+/}Ncr1^{cre+}$, termed HIF-2α KO). In this experimental setting we failed to detect any effects on splenic NK cell numbers or NK cell activation upon stimulation PMA/ionomycin under normoxia (20% O_2) and hypoxia (2% O_2) (Fig. 3g and h), suggesting HIF-1α as the main mediator of hypoxic responses in NK cells. However, it is important to mention that VHL targets several proteins outside the HIF pathway for ubiquitination and degradation, e.g. members of the p53 and NF-κB pathway as well as atypical protein kinase C and RNA polymerase II [35]. Therefore, we can not entirely rule out that loss of VHL also modulates HIF-independent cellular signaling events in NK cells.

2. The authors have described defects in degranulation in response to non-specific stimuli (PMA/ionomycin) but have not shown impaired cytotoxicity of tumor cells directly. It is possible that cytotoxicity is more profoundly affected or is unaffected, which would also be interesting! If cytotoxicity is unaffected, it might suggest infiltration into the hypoxic tumor microenvironment might be part of the mechanism here.

OUR REPLY:

We appreciate this comment and would like to point out that our previous report (Krzywinska et al. 2017; PMID: 29150606; Fig. 1 and Suppl. Figure 1) demonstrates impaired *in vitro* killing (of MHC-I-deficient lymphoma cells) by HIF1a-deficient NK cells. Therefore, we now performed additional *in vitro* killing assays with B16 melanoma cells. Spontaneous killing of B16 melanoma cells by NK cells in a standard killing assay (for 6 h at a 10:1 effector/target ratio without cytokines was only about 2%, data not shown). So, we customized the killing assay for B16 cells by increasing the coincubation to 24 hours and the addition of IL-15 to ensure NK cell survival over this period. However, as shown below, the loss of HIF1a did not affect NK cell-dependent lysis of B16 cells in normoxia or hypoxia.

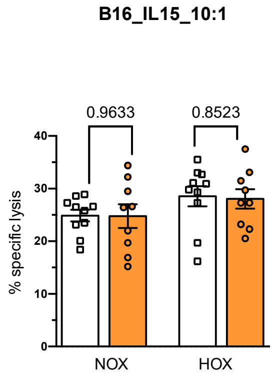

This is in line with previous reports from our lab that HIF1a does not affect the in vitro killing of MHC-I-proficient cells (Krzywinska et al. 2017). Hence, given the profound effect of HIF1a on NK cell-dependent pulmonary metastasis in vivo, we decided to analyze abundance and phenotype of NK cells in lungs from mice after i.v. injection of B16 melanoma cells.

As shown below, the increase in pulmonary metastasis HIF1a KO mice is associated with a decrease in pulmonary NK cell frequencies:

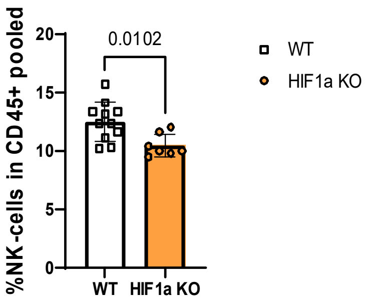

This consistent with previous reports from our lab, where we have shown that loss of HIF1a leads to impaired NK cell infiltration in vivo (Krzywinska et al. 2017).

This data is now included in the new Figure 3 of the revised manuscript and described as follows:

NK cells have a unique capacity to restrict metastatic spread of circulating cancer cells[8]. Therefore, we aimed to determine the impact of HIF-1α expression on NK cell effector function and immunosurveillance of metastatic tumor cells *in vivo*. To this end, we injected syngeneic B16F10 melanoma cells intravenously into HIF-1α KO mice as well as the

corresponding WT littermates and analyzed the number of pulmonary metastases 2 weeks post-injection. Consistent with our *in vitro* observations, loss of HIF-1α in NK cells leads to increased pulmonary metastasis of intravenously injected melanoma cells (Fig. 3i). Given the profound effect of HIF1a on NK cell-dependent pulmonary metastasis in vivo, we decided to analyze abundance and phenotype of NK cells in lungs from mice after i.v. injection of B16 melanoma cells. As shown in Figure 3f, the increase in pulmonary metastasis HIF1a KO mice is associated with a decrease in pulmonary NK cell frequencies and total numbers, along with a decrease in pulmonary Granzyme B+ and IFN-γ + NK cells. This is consistent with previous reports from our lab, where we have shown that loss of HIF1a leads to impaired NK cell infiltration in vivo [20]

Next, we now performed in vitro killing assays with B16 melanoma cells. Spontaneous killing of B16 melanoma cells by NK cells in a standard killing assay (for 6 h at a 10:1 effector/target ratio) without cytokines was only about 2% (data not shown). So, we customized the killing assay for B16 cells by increasing the coincubation to 24 hours and the addition of IL-15 to ensure NK cell survival over this period. However, as shown below, the loss of HIF1a did not affect NK cell-dependent lysis of B16 cells in normoxia or hypoxia (Fig. 3g). This is in line with previous reports from our lab that HIF1a does not affect the in vitro killing of MHC-I-proficient cells [20] and suggests that HIF-1α is required NK cell infiltration into metastatic lesions.

Next, we analyzed the abundance and phenotype of NK cells in lungs from VHL KO mice after i.v. injection of B16 melanoma cells.

As shown below, the decrease in pulmonary metastasis in VHL KO mice is associated with an increase in Granzyme B+ NK cells, whereas the frequency of total NK cells remained unchanged:

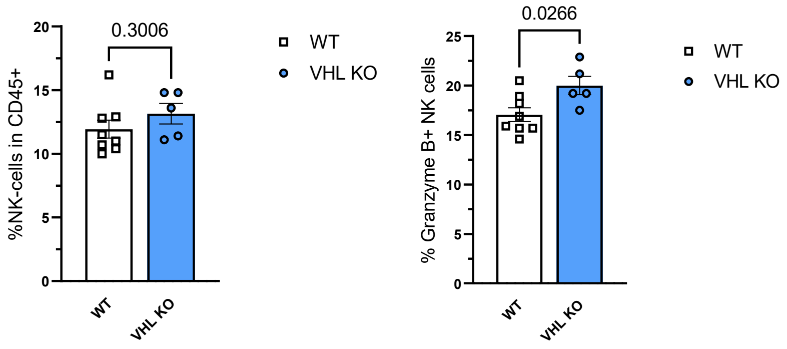

This data is now included in the new Figure 4 of the revised manuscript and described as follows:

Next, we aimed to determine the impact of VHL loss on NK cell immunosurveillance of metastatic tumor cells *in vivo* and injected syngeneic B16F10 melanoma cells intravenously into VHL KO mice as well as the corresponding WT littermates and analyzed the number of pulmonary metastases 2 weeks post-injection. Consistent with our *in vitro* observations, VHL deletion in NK cells reduced pulmonary metastases by almost 70% (Fig. 4g). Furthermore, this was associated with with an increase in Granzyme B+ NK cells, whereas the frequency of total NK cells remained unchanged. (Fig. 4h). This suggests that forced HIF-1α-driven glycolysis can enhance NK cell effector function *in vivo*.

3. The histological features of the tumor and lack or not of NK cell penetrance into the tumor has not been addressed here either. NK cell infiltration studies would be an interesting further dissection of this model. At the very least, these limitations of the study should be acknowledged.

OUR REPLY:

We appreciate this comment and would like to refer to our reply to the previous comment just above.

4. Some comment should be made on how the authors believe such a robust effect is seen in vivo, from remarkably subtle changes in apoptosis and degranulation.

OUR REPLY:

The reviewer mentions a critical point that we appreciate. PMA/ionomycin stimulation is well accepted and commonly used in the field. But indeed PMA/ionomycin is a chemical and, hence rather artificial stimulus, compared to more physiological e.g. ligand-mediated activation. Therefore, this harsh way of maximal NK cell activation likely masks more subtle but eventually physiologically still relevant activation defects in HIF1a KO NK cells. In contrast, target cells that NK cells encounter *in vivo*, express a variety of ligands (activating as well as inhibitory) and therefore represent a more physiological setting of NK cell activation. Moreover, *in vivo*, target cells and NK cells compete for nutrients and oxygen within their microenvironment. Therefore, the absence of HIF1a is presumably more deleterious for NK cells *in vivo* and the negative impact on NK cell performance more profound.

5. One of the most interesting points of this study is that it conflicts with the report by Ni et al. Immunity 2020 (10.1016/j.immuni.2020.05.001). The authors should discuss why they think their studies differ from those of Ni et al. to give a perspective on the differences.

OUR REPLY:

We very much appreciate this comment. Although, we can not exhaustively clarify all the contradictions between our studies and the report by Ni et al., we would like to propose some explanations for the obvious discrepancies. One likely explanation is the use of different tumor models and, hence, target cells. We used in this and previous studies YAC-1 and v-ABL lymphoma cell lines as well LLC lung carcinoma, MC 30 colon cancer and B16F10 melanoma cell lines for in vivo studies. Ni et al. mostly studied subcutaneous RMA-s and RMA tumors and in one experiment LLC tumors with different initial numbers of subcutaneously injected tumor cells. Of note, Ni et al. did not perform intravenous injection of B16 cells as we did here, to selective study the impact of HIF1a on NK cell surveillance of circulating melanoma cells.
Additional confounding factors could be the degree of backcrossing to the BL6 background (in our case > 99% C57Bl/6J background) as well as varying hygiene

levels in different animal husbandry and the potential consequences on preactivation of NK cells.

Besides that, Ni et al performed all *ex vivo* Seahorse analysis under normoxic conditions and not in hypoxia. Yet, Ni et al. also observe increased OxPhos in HIF1a deficient NK cells in Seahorse. However, whether increased OxPhos affects ROS production or DNA damage was not evaluated further.

Finally, in contrast to our studies, Ni et al. did not observe significant differences in splenic NK cell numbers (Suppl. Fig 1 C of their publication). Although, there was a trend towards decreased NK. cell numbers in HIF1a KO mice, with an n = 3, this trend failed to reach the level of statistical significance.

Although, we can not entirely reconcile our results with the report by Ni et al., the above-mentioned differences might explain some of the contradictions.

6. There are some important omissions in the methods section. What are the details of the B16F10 mouse model? How many tumor cells were injected per animal? How was the granzyme B measured following PMA/ionomycin stimulation performed? Is this intracellular or secreted granzyme B? Providing these details would allow a more in depth discussion of how this manuscript differs from the models put forward by Ni et al.

OUR REPLY:

We apologize for this oversight, the "methods" section of the revised manuscript now contains a more detailed description of the requested experimental details as follows:

Mouse models. *In vivo*, targeted deletions of HIF-1α and VHL in NK cells were achieved by as previously described (Krzywinska et al., 2017, 2022). To mitigate the influence of strain variation, mice were kept in a > 99% C57Bl/6J background. Male and female mice at the age of 8 to 12 weeks were used at equal ratios.

Pulmonary metastasis model. B16F10 melanoma cell line was culture in a RPMI GlutaMAX TM medium (Thermofisher) supplemented with 10% of fetal bovine serum (FBS) and 50 U/mL penicillin-streptomycin at 37°C and 5% CO_2. Cells were resuspended in PBS and 1 x

10^5 (for HIF1α mouse model) or 2 x 10^5 (for VHL mouse model) B16F10 cells in 50 µL of PBS were injected into the tail vein of the animals. The lungs were removed after 7 days for NK cell infiltration assessment and stimulation assay, and after 14 days for metastatic foci counting.

NK cell purification. Splenocytes were obtained from HIF-1α WT and KO mice. NK cells were purified on ice using “EasySep™ Mouse NK Cell Isolation Kit” following manufacturer’s protocol.

Stimulation assay. Splenocytes were prepared and cultured with PMA (20 ng/mL) and ionomycin (1µg/mL) in a complete RPMI medium for 6 hours at 37°C in normoxia (20% O2) or hypoxia (1% O2). Golgi Stop (BD), Golgi Plug (BD) and anti-CD107a were added in the medium at the beginning of stimulation. Surface and intracellular staining for granzyme B and INF-γ were performed.

7. There are also a number of typos in the document that should be corrected e.g. 'cytoxic' rather than 'cytotoxic' in the introduction.

OUR REPLY:

We apologize for this and have now screened and corrected the manuscript for typos.

8. I believe Figure 2i is mislabeled in the legend.

OUR REPLY:

We apologize for this mistake and have now corrected the legend of the corresponding figure.

Referee #3:

The report by Pelletier et al. describes the in vivo consequences of HIF loss-of-function or gain-of-function mutations in resting and activated NK cells using conditional knockout mice. In addition to providing evidence in support of a role of HIF-1 in activated NK cells residing in a hypoxic environment, the authors make a novel link of HIF-1 signaling with tryptophan and nicotinamide metabolism in resting NK cells, which has functional consequences on OxPhos-associated mitochondrial ROS production, DNA damage, and apoptosis. The data include global untargeted metabolomic and transcriptomic measurements of rapidly purified, resting NK cell-enriched cell populations from spleens, which revealed a decrease in tryptophan and NAM levels as well as in mRNA levels for cellular proteins involved in tryptophan uptake and catabolism, respectively. Targeted metabolomics revealed reduced tryptophan catabolites and NAD precursors as well as a decreased free NAD+/NADH ratio. The authors also report oxygen consumption rate (OCR), fatty acid oxidation, and glutaminolysis data for splenic cells isolated from WT or HIF-1a NK-cell conditional KO mice and perform a subset of these studies with NK cells isolated from NK-cell conditional VHL knockout mice.

A major strength of this study is a focus on the in vivo aspect of NK biology using genetically modified mice, which is the most physiologically relevant model for these studies. The authors use floxed HIF-1a and HIF-2a mice as well as floxed VHL conditional mice in conjunction with an NK cell-restricted Ncr1-Cre driver transgene to examine the effect of impaired or augmented HIF signaling, respectively, in splenic NK cells. The studies are generally robust and the conclusions reasonable. However, there are areas in which the current study could be strengthened. My concerns for this study are as follows.

OUR REPLY:

We appreciate the overall positive reception of our work and the very constructive comments.

Figure 1:

• The differences in gene expression data in Figure 1d are a bit difficult to appreciate plotted only in this manner. It may be helpful to add another panel where you normalize each gene to 100% for the WT level and compare the KO in this manner (relative gene expression patterns).
• Moreover, the criteria for "statistical significance" with $P>0.05$ may be too stringent for this small sample size. I suspect that Kmo and Kynu levels may reach a significance of $P>0.10$, which would be further supported by the targeted metabolomic data in Fig 1e.
• It appears that Haao levels may have reached the $P>0.05$ threshold. If so, this should be so designated and the implications commented on.
• It would be helpful to know what gene expression levels are for Qprt, the enzyme catalyzing conversion of quinolinic acid (QA) to NAD.

OUR REPLY:

We appreciate this comment and created panels displaying absolute values and relative gene expression patterns including the gene Qprt with $P>0.10$ as criteria for statistical significance :

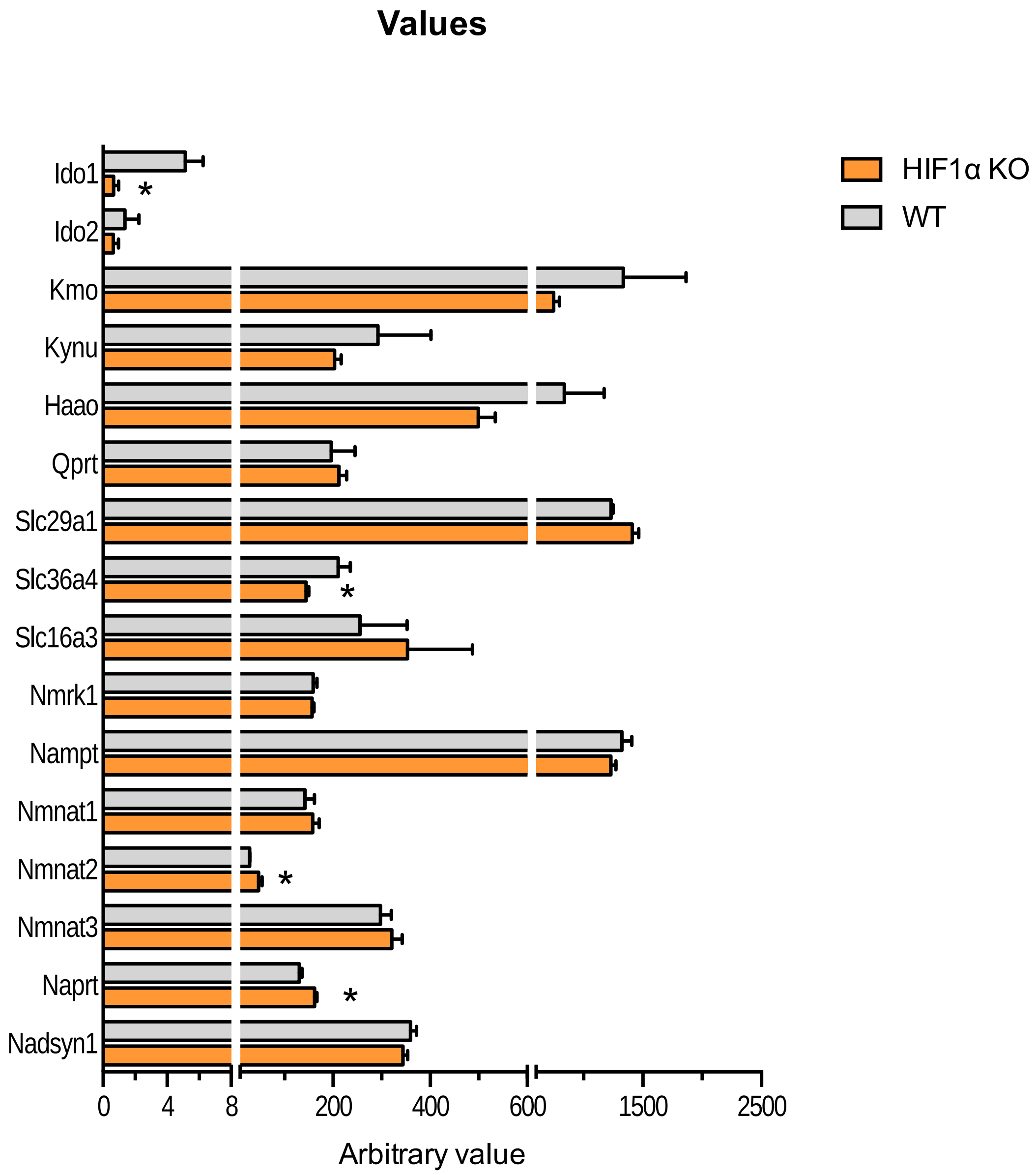

Values
HIF1α KO
WT
Ido1
Ido2
Kmo
Kynu
Haao
Qprt
Slc29a1
Slc36a4
Slc16a3
Nmrk1
Nampt
Nmnat1
Nmnat2
Nmnat3
Naprt
Nadsyn1
0
4
8
200
400
600
1500
2500
Arbitrary value

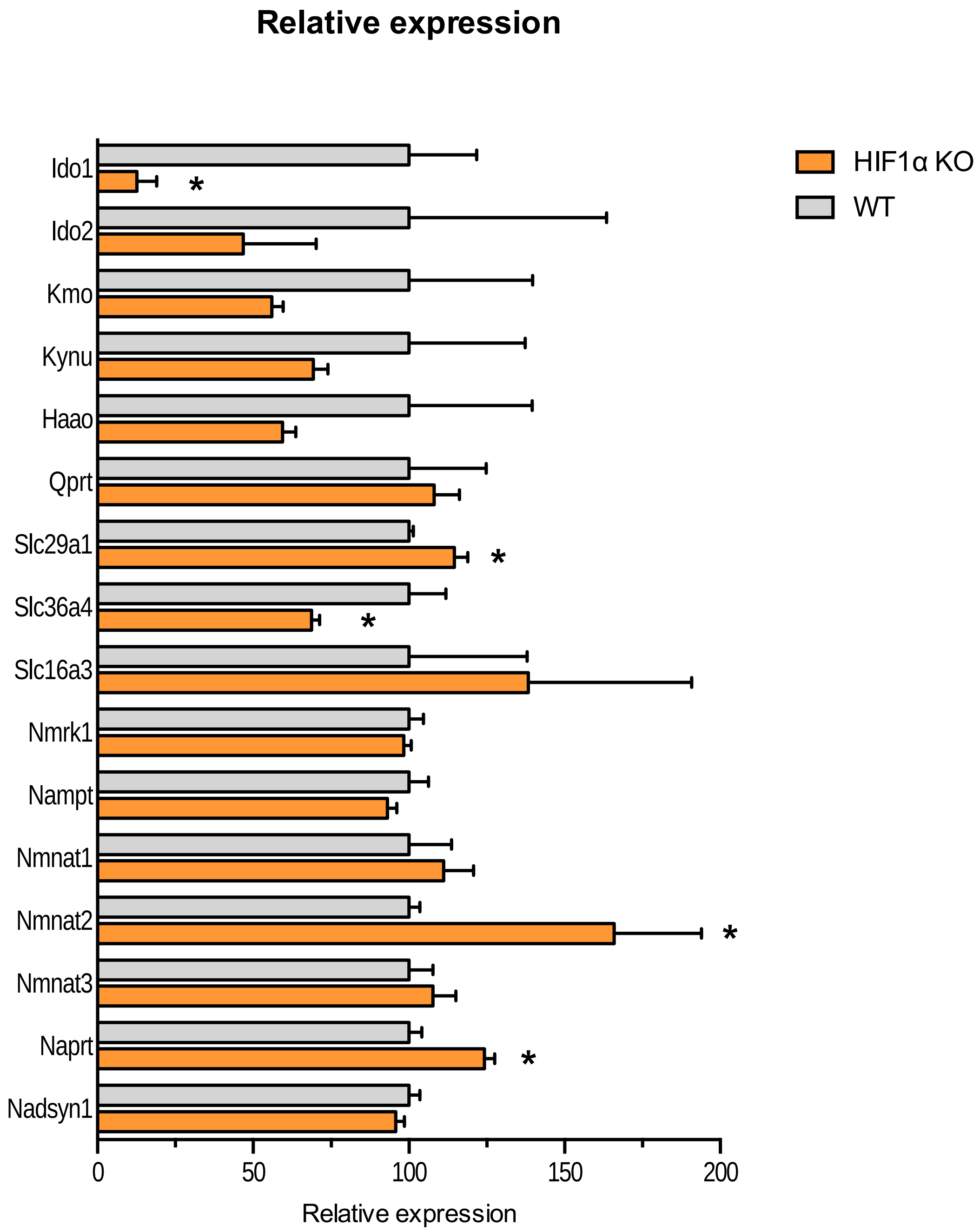

We would like to leave it to the reviewers and the editor which panel should replace the initial panel of the figure or whether we should keep both data representations.

• In view of the above, it appears that there may be a pathway decrease in synthetic enzymes involved in QA production, consistent with how HIF-1 regulates glycolytic enzymes. This may have important implications outside of this study. A pathway-type analysis should be performed to test this hypothesis (see below for Figure 2h).

OUR REPLY:

We appreciate this comment and performed GSEA for all enzymes involved in QA production. As shown below, this revealed an enrichment in WT NK cells and a downregulation in HIF1a-deficient NK cells for genes involved in the tryptophan - quinolinic acid pathway:

	SYMBOL	RANK IN GENE LIST	RANK METRIC SCORE	RUNNING ES	CORE ENRICHMENT
1	Acmsd	5333	0.259	-0.1145	No
2	Kyat1	7548	0.161	-0.1036	No
3	Kyat3	8006	0.144	-0.0220	No
4	Afmid	11165	0.022	-0.0490	No
5	Tdo2	12420	-0.011	0.0005	No
6	Ido2	19959	-0.353	-0.2027	Yes
7	Kynu	20435	-0.391	-0.1218	Yes
8	Haao	22060	-0.504	-0.0872	Yes
9	Kmo	22615	-0.550	-0.0094	Yes
10	Ido1	24807	-1.794	0.0024	Yes

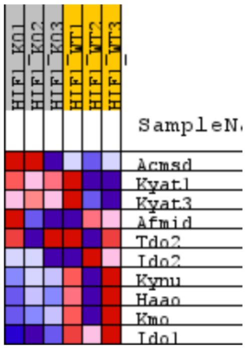

• For Figure 1e, if this information is available, it would be helpful to know levels of other metabolites in this pathway besides KYN that are measurable by targeted metabolomics (3-HK, 3-HAA, QA). This may be of particular importance to the oxidative stress assessment since several of these metabolites may have pro- or anti-oxidant effects in other settings.

OUR REPLY:

We appreciate this suggestion and would like to point out that the initial manuscript contained all measurable metabolites with a significantly different abundance between genotypes. Unfortunately, 3-HK and 3-HAA were not part of the analysis but we now included the QA measurement (see below) in the new Suppl. Figure 1e of the revised manuscript. The metabolomics data are deposited on the Metabolights platform (https://www.ebi.ac.uk/metabolights/login), email address (abigaelle.pelletier@anatomy.uzh.ch) and password (Anatomy22).

e

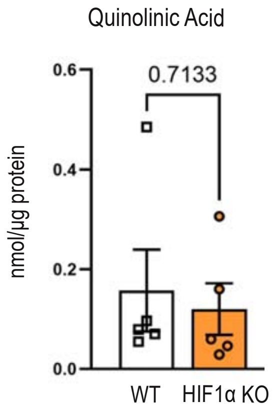

• To assist scientists outside this field, designations for all genes and proteins in Figure 1e should be clarified in the legend.

<u>OUR REPLY:</u>

We apologize for this oversight and the designations are now included in the corresponding figure legends as follows:

Figure 1:

a. NK cell count, defined as CD45+ CD3- NKp46+ CD49b+, in spleens of WT and HIF-1α KO mice (n=2). **b.** Experimental design for *in situ* experiments. **c.** Heatmap of the down-regulated metabolites from the untargeted metabolomics performed on 6 samples of freshly isolated NK cells from WT and HIF-1α KO mice. **d.** Gene expression analysis of genes from the NAD – tryptophan pathway and key amino acid transporters from bulk RNA-sequencing performed on 6 samples of freshly isolated NK cells from WT and HIF-1α KO mice. **e.** The NAD – tryptophan pathway (NAAD, nicotinic acid adenine dinucleotide; NA, nicotinic acid; NAMN, nicotinic acid mononucleotide; NMN, nicotinamide mononucleotide; NAM, nicotinamide; MNA, methyl-nicotinamide; NAR, nicotinic acid riboside; NAD, nicotinamide adenine dinucleotide; NADP, NAD phosphate; IDO, indoleamine-2,3-dioxygenase; KMO, kynurenine 3-monooxygenase; KYNU, kynureninase; HAAO, 3-hydroxyanthranilate 3,4-dioxygenase; QPRT, quinolinate phosphoribosyl-transferase; NMNAT, NMN adenyl-transferase; NAPRT, NA phosphoribosyl-transferase; NADS, NAD synthase; NAMPT, NAM phosphoribosyl-transferase; NRK, NR kinase) and analysis of

metabolites quantification from untargeted (tryptophan, methyl-quinoline and NAM) and targeted (NAAD, NA, NMN, MNA and kynurenine) metabolomics.

• It is important to acknowledge that the NAD/NADH ratio reported in Figure 1f is based upon total NAD and NADH levels, whereas it is free NAD+ and NADH that has regulatory roles. This is a limitation that is frequently not acknowledged.

OUR REPLY:

We appreciate this remark and changed the text of the revised manuscript as follows:

While the mRNA expression of enzymes that convert NAD precursors into NAD was not affected (Fig. 1d), HIF-1α-deficient cells had a decreased NAD/NADH ratio (Fig. 1f). Although, the quantification of NAD/NADH ratio here is based on total NAD and NADH, this suggests a so far unanticipated role of HIF-1α in tryptophan metabolism and the maintenance of free NAD^+ levels in resting, non-activated NK cells.

Figure 2:

• In line with additional measurements of gene expression detailed above for Figure 1d, a GSEA would be helpful for the tryptophan/quinolinic acid biosynthetic pathway outlined in Figure 1e.

OUR REPLY:

We appreciate this comment and performed GSEA for all enzymes involved in QA production. As shown below, this revealed an enrichment in WT NK cells and a downregulation in HIF1a-deficient NK cells for genes involved in the tryptophan - quinolinic acid pathway:

	SYMBOL	RANK IN GENE LIST	RANK METRIC SCORE	RUNNING ES	CORE ENRICHMENT
1	Acmsd	5333	0.259	-0.1145	No
2	Kyat1	7548	0.161	-0.1036	No
3	Kyat3	8006	0.144	-0.0220	No
4	Afmid	11165	0.022	-0.0490	No
5	Tdo2	12420	-0.011	0.0005	No
6	Ido2	19959	-0.353	-0.2027	Yes
7	Kynu	20435	-0.391	-0.1218	Yes
8	Haao	22060	-0.504	-0.0872	Yes
9	Kmo	22615	-0.550	-0.0094	Yes
10	Ido1	24807	-1.794	0.0024	Yes

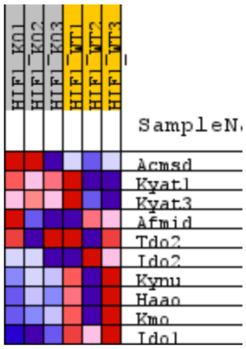

• For improved clarity, may be best to state the legend for Figure 2k as "Same as (i) compared to supplementation with 10 mM NAD+ or NAM (n=2)."

OUR REPLY:

We have revised the figure legend accordingly.

Figure 3:
• To confirm the extent of Cre-mediated excision, immunoblots of VHL and HIF-2, (and HIF-1 in Figure 1), protein levels in purified NK cells isolated from HIF-1, HIF-2, or VHL mice and maintained under normoxic or hypoxic conditions should be performed.
• The above is particularly important as the floxed HIF-2 allele following Cre excision reportedly generates a HIF-2 protein that lacks the bHLH domain, but retains the rest of the HIF-2 protein. Thus, this floxed HIF-2 mouse may not generate a true null allele following Cre-mediated excision and instead may be a potential hypomorph allele, which may or may not be deficient in HIF-2 mediated effects. If this floxed HIF-2 allele generates a HIF-2 protein that is detectable by a polyclonal antibody recognizing the carboxy terminus, this should be acknowledged and the above limitations stated.

OUR REPLY:

We appreciate this very important point raised by the reviewer. We would like to point out that our previous publication demonstrated efficient deletion of HIF1a at the mRNA and protein level in splenic NK cells from HIF1a KO mice in hypoxia (Krzywinska et al. 2017) as well increased HIF1a protein expression in NK cells from VHL KO mice in normoxia (Sobecki and Krzywinska et al. 2021). We are aware of the problem that different floxed HIF-2 mice circulate within the community and that some founders do not yield full excision but rather a truncated version of HIF2a. Therefore, we have initially obtained the floxed HIF-2 allele from collaborators that have thoroughly validated the HIF2a knockout at the protein level (https://www.ncbi.nlm.nih.gov/pmc/articles/PMC5698059/) in order to generate our NK cell-specific knockout mice. However, if the reviewers and the editor consider this data as not solid enough, we would like to offer to remove the HIF2 results from the manuscript.

• The investigators conclude from the data with HIF-2 floxed mice in Figure 3h that Cre-mediated deletion of the floxed exon does not affect the FACS profiling defined by CD107a, IFN-g, or GrzmB detection. However, the sample size for some of the experiments, particularly the hypoxic ones, was much smaller than that defined by CD107a and IFN-g profiling for HIF-1 floxed mice in Figure 2j, and as such may not detect a true difference if there is a smaller effect size. Accordingly, the p-value for the comparisons in Figure 3h should be presented in the figure legend to allow the reader to judge whether this result is significant or not, rather than just deemed non-significant. This approach would be best for all data presented in this study.

<u>OUR REPLY:</u>

We appreciate this comment and provide now the p-value for each graph (see below and new Suppl. Figure 2):

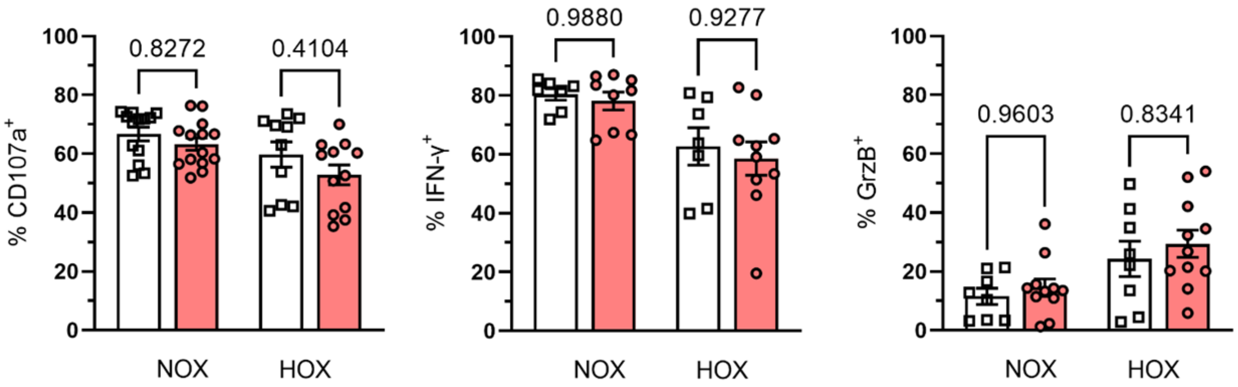

• GrzmB profiling was not shown for the NK cells from HIF-1 floxed mice. If performed, it should be shown even if similar as data generated from WT mice.

<u>OUR REPLY:</u>

We appreciate this suggestion and agree with the reviewer that information on GrzmB expression in HIF1a KO NK cells is helpful. As shown below, in contrast to CD107a and IFNg, GrzmB levels are not reduced in HIF1a KO NK cells after PMA/ionomycin stimulation for 6 hours. However, we would like to point out that kinetics for CD107A as a degranulation marker and GrzmB are different.

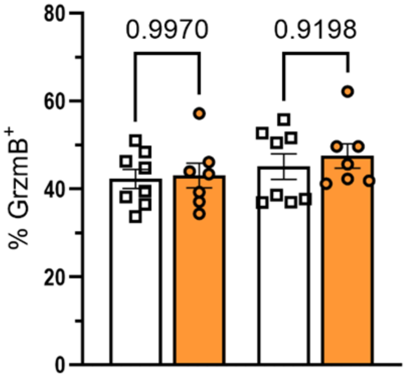

• Although the VHL null results are highly suggestive, it is unclear why more was not done to clarify the respective in vivo roles for HIF-1 versus HIF-2 using this model. Specifically, use of a VHL/HIF-1 and VHL/HIF-2 doubly floxed mice with the Ncr1-Cre driver would test the contributions of HIF-1 versus HIF-2, respectively, in the VHL knockout phenotype. I recognize that this may be the basis for future experiments.

OUR REPLY:

We agree that VHL/HIF-1 and VHL/HIF-2 double KO mice would be very useful to dissects out distinct contributions of HIF1 and HIF2, respectively. We are indeed currently generating such mouse strains. However, the time to perform all the experiments in both strains by the time we expect the obtain the mouse strains with full KO of both alleles would be beyond the timeline of a revision at EMBO reports.

Dear Prof. Stockmann,

Thank you for submitting your revised manuscript for consideration by EMBO reports. We have now received the full set of comments from the three referees that were asked to re-evaluate your study. Please find their comments included below.

As you will see, all referees find that the study was substantially improved during revision and that most of their concerns were successfully addressed. Referees #1 and #3 request only few minor changes in the text and figures, which I need you to address before I can accept the manuscript. Please make sure that all changes are highlighted to be clearly visible in the revised manuscript file.

From the editorial side, there are a number of things that we need from you:

- Please change the heading "Summary" to "Abstract".

- Please provide up to 5 keywords in your revised manuscript (you currently have 6).

- According to our journal's policy, "data not shown" (stated on page 7 of your manuscript) is not permitted. All data referred to in the paper should be displayed in the main or Expanded View figures, or in the Appendix. Please add these data or change the text accordingly if these data are not central to the study and its conclusions.

- For better structuring of your Results and discussion section, please provide a sub-heading for the first part.

- Your Figure legends have been inspected by our data editors for completeness and accuracy. Please see the required changes in the attached Word file and address all comments in your revised manuscript (with tracked changes).

- Figure callouts for Fig 4A are missing, please make sure that all panels are called out in your revised manuscript.

- We have replaced Supplementary Information with Expanded View (EV) Figures that are collapsible/expandable online. We recommend that you promote your two "Appendix Figures" to the Expanded View format by renaming "Appendix Figure 1" and "Appendix Figure 2" to "Figure EV1" and "Figure EV2", respectively. EV Figures should be cited as "Figure EV1" or "Figure EV2" in the text and their respective legends should be included in the main text after the legends of main figures, following the heading "Expanded View Figure legends".

- "Methods" needs correcting to "Materials and Methods".

- Please update your Data availability statement: the heading should be "Data availability" (placed after Materials and Methods), and it should follow the example/model below:

"
Data availability

The datasets produced in this study are available in the following databases:

- RNA-Seq data: Gene Expression Omnibus GSE46843 (https://www.ncbi.nlm.nih.gov/geo/query/acc.cgi?acc=GSE46843)
- [data type]: [name of the resource] [accession number/identifier/doi] ([URL or identifiers.org/DATABASE:ACCESSION])
"

* Please note that all datasets should be publicly available at the time of publication, and therefore no passwords are necessary in the Data availability statement.

- We noted that grant # 310030_212504 is missing from your Acknowledgments statement in the manuscript.

- Please update your competing interests statement: the heading should be "Disclosure and competing interests statement" and the statement "The authors declare that they have no conflict of interest.", since you have no competing interests to declare.

- The author contributions statement should be removed from the manuscript file. Instead, we now use CRediT to specify the contributions of each author in the journal submission system. Please use the free text box to provide more detailed descriptions. See also our guide to authors:
<https://www.embopress.org/page/journal/14693178/authorguide#authorshipguidelines>.

- You are kindly requested to note our reference format (> 10 authors are listed) and to update the list of references accordingly:

<http://www.embopress.org/page/journal/14693178/authorguide#referencesformat>.

- The manuscript information at the top of the Author Checklist has not been completed, please upload the completed checklist.

- Please note that all source data for the main figures should be uploaded in one ZIP file, and all source data for the Expanded View (previously "Appendix") figures in another.

- Thank you for uploading a synopsis image. Please note that all text needs to be readable at the final size (550 pixels wide, the height is variable). Could you please change the font sizes as necessary and re-submit the image?

- Please note that EMBO press papers are accompanied online by
A) a short (1-2 sentences) summary of the findings and their significance, and
B) 2-4 short bullet points highlighting the key results
Please send us this information along with your revised manuscript.

Please also note that as part of the EMBO publications' Transparent Editorial Process, EMBO reports publishes online a Review Process File to accompany accepted manuscripts. This File will be published in conjunction with your paper and will include the referee reports, your point-by-point response and all pertinent correspondence relating to the manuscript.

You can opt out of this by letting the editorial office know (emboreports@embo.org). If you do opt out, the Review Process File link will point to the following statement: "No Review Process File is available with this article, as the authors have chosen not to make the review process public in this case."

We would also welcome the submission of cover suggestions or motifs to be used by our Graphics Illustrator in designing a cover.

We look forward to seeing a final version of your manuscript as soon as possible.

Yours sincerely,

Ioannis Papaioannou, PhD
Editor
EMBO reports

Referee #1:

In the point-to-point response and the revised version of the manuscript the authors have addressed my concerns sufficiently. However, I would find it helpful if the authors could add a short paragraph to the actual manuscript describing the potential reasons for their partial contradictive tumor model results with the Krzywinska et al. 2017 and Ni et al. 2020 studies (different tumor models etc.; as they explain it in the point-to-point response to reviewers).

Referee #2:

The authors have addressed all of our comments. Thank you

Referee #3:

In this revised report, the investigators have made substantial improvements that address most of my prior concerns. Here are my comments/concerns relating to the revised study.

Figure 1:
• The relative difference in qPCR results provide a clearer perspective for assessing differences in gene expression for these

data. I favor using the relative difference exclusively in the main figure and providing absolute values as supplemental data (although latter not required).

• The GSEA for QA production that the investigators have constructed is very informative. It should be included in supplemental data along with a pathway showing positions of the genes/proteins involved in QA production reported in this GSEA.

• Some of the abbreviations in the diagram and legend for the tryptophan/NAD pathway metabolites do not match (NR and NAR). The authors should edit one or the other for consistency, then adjust text and legend once a uniform designation has been made.

• As it currently stands, there are missing designations for both metabolites (Trp, Kyn, QA), plus there are designations for metabolites/cofactors (NADP) and enzymes (QPRT) that are not indicated in the pathway. Attention to detail for this valuable visual aid would be helpful, even if levels of metabolites or of mRNA encoding enzymes were not determined. Also, it may be best to define all metabolite abbreviations in a first sentence and all enzyme abbreviations in a second sentence.

Figure 2:

• Similar comments as for Figure 1 regarding the value of the GSEA and its inclusion in supplemental data.

Appendix Figure 1:

• The abbreviation in the legend and in the figure do not match.

Appendix Figure 2:

• The author's response to concerns raised about data in the previous Figure 3h cites a reference for a presumptive second founder for the floxed HIF-2 strain. The problem is not with use of different founders, but with the possible initiation of a truncated HIF-2 protein from the transcript made from the Cre-excised HIF-2 locus. Unless the immunoblots demonstrate that this smaller protein is absent following Cre excision, the investigators should recognize that a hypomorphic or transdominant negative form of HIF-2 protein may be produced following Cre excision. If produced, its presence may compromise, or even invalidate, conclusions of all studies using this floxed strain if investigators incorrectly assume that the Cre-excised allele is a null allele.

Referee #3:

In this revised report, the investigators have made substantial improvements that address most of my prior concerns. Here are my comments/concerns relating to the revised study.

Figure 1:
• The relative difference in qPCR results provide a clearer perspective for assessing differences in gene expression for these data. I favor using the relative difference exclusively in the main figure and providing absolute values as supplemental data (although latter not required).

• The GSEA for QA production that the investigators have constructed is very informative. It should be included in supplemental data along with a pathway showing positions of the genes/proteins involved in QA production reported in this GSEA.

Reply: Done!

• Some of the abbreviations in the diagram and legend for the tryptophan/NAD pathway metabolites do not match (NR and NAR). The authors should edit one or the other for consistency, then adjust text and legend once a uniform designation has been made.

Reply: Done!

• As it currently stands, there are missing designations for both metabolites (Trp, Kyn, QA), plus there are designations for metabolites/cofactors (NADP) and enzymes (QPRT) that are not indicated in the pathway. Attention to detail for this valuable visual aid would be helpful, even if levels of metabolites or of mRNA encoding enzymes were not determined. Also, it may be best to define all metabolite abbreviations in a first sentence and all enzyme abbreviations in a second sentence.

Reply: Done!

Appendix Figure 1:
• The abbreviation in the legend and in the figure do not match.

Reply: Done!

Appendix Figure 2:
• The author's response to concerns raised about data in the previous Figure 3h cites a reference for a presumptive second founder for the floxed HIF-2 strain. The problem is not with use of different founders, but with the possible initiation of a truncated HIF-2 protein from the transcript made from the Cre-excised HIF-2 locus. Unless the immunoblots demonstrate that this smaller protein is absent following Cre excision, the investigators should recognize that a hypomorphic or transdominant negative form of HIF-2 protein may be produced following Cre excision. If produced, its presence may compromise, or even invalidate, conclusions of all studies using this floxed strain if investigators incorrectly assume that the Cre-excised allele is a null allele.

Reply: We have added a sentence to the manuscript that mentions the fact that a hypomorphic or transdominant negative form of HIF-2 protein may be produced following Cre excision.

Prof. Christian Stockmann
University of Zurich
Winterthurerstrasse 190
Zurich 8057
Switzerland

Dear Prof. Stockmann,

I am very pleased to accept your manuscript for publication in the next available issue of EMBO reports. Thank you for your contribution to our journal.

At the end of this email I include important information about how to proceed. Please ensure that you take the time to read the information and complete and return the necessary forms to allow us to publish your manuscript as quickly as possible.

As part of the EMBO publication's Transparent Editorial Process, EMBO reports publishes online a Review Process File to accompany accepted manuscripts. As you are aware, this File will be published in conjunction with your paper and will include the referee reports, your point-by-point response and all pertinent correspondence relating to the manuscript.

If you do NOT want this File to be published, please inform the editorial office within 2 days, if you have not done so already, otherwise the File will be published by default [contact: emboreports@embo.org]. If you do opt out, the Review Process File link will point to the following statement: "No Review Process File is available with this article, as the authors have chosen not to make the review process public in this case."

Should you be planning a Press Release on your article, please get in contact with emboreports@wiley.com as early as possible, in order to coordinate publication and release dates.

Thank you again for your contribution to EMBO reports and congratulations on a successful publication. Please consider us again in the future for your most exciting work.

Yours sincerely,

Ioannis Papaioannou, PhD
Editor
EMBO reports

****

THINGS TO DO NOW:

Please note that you will be contacted by Wiley Author Services to complete licensing and payment information. The required 'Page Charges Authorization Form' is available here: https://www.embopress.org/pb-assets/embo-site/er_apc.pdf - please download and complete the form and return to embopressproduction@wiley.com

EMBO Press participates in many Publish and Read agreements that allow authors to publish Open Access with reduced/no publication charges. Check your eligibility: https://authorservices.wiley.com/author-resources/Journal-Authors/open-access/affiliation-policies-payments/index.html

You will receive proofs by e-mail approximately 2-3 weeks after all relevant files have been sent to our Production Office; you should return your corrections within 2 days of receiving the proofs.

Please inform us if there is likely to be any difficulty in reaching you at the above address at that time. Failure to meet our deadlines may result in a delay of publication, or publication without your corrections.

All further communications concerning your paper should quote reference number EMBOR-2022-56156V3 and be addressed to emboreports@wiley.com.

Should you be planning a Press Release on your article, please get in contact with emboreports@wiley.com as early as possible, in order to coordinate publication and release dates.

EMBO Press Author Checklist

Corresponding Author Name: Christian Stockmann	
Journal Submitted to: EMBO Reports	
Manuscript Number: EMBOR-2022-56156V2	

USEFUL LINKS FOR COMPLETING THIS FORM
The EMBO Journal - Author Guidelines
EMBO Reports - Author Guidelines
Molecular Systems Biology - Author Guidelines
EMBO Molecular Medicine - Author Guidelines

Reporting Checklist for Life Science Articles (updated January 2022)
This checklist is adapted from Materials Design Analysis Reporting (MDAR) Checklist for Authors. MDAR establishes a minimum set of requirements in transparent reporting in the life sciences (see Statement of Task: 10.31222/osf.io/9sm4x). Please follow the journal's guidelines in preparing your manuscript.
Please note that a copy of this checklist will be published alongside your article.

Abridged guidelines for figures
1. Data
The data shown in figures should satisfy the following conditions:
- the data were obtained and processed according to the field's best practice and are presented to reflect the results of the experiments in an accurate and unbiased manner.
- ideally, figure panels should include only measurements that are directly comparable to each other and obtained with the same assay.
- plots include clearly labeled error bars for independent experiments and sample sizes. Unless justified, error bars should not be shown for technical replicates.
- if n<5, the individual data points from each experiment should be plotted. Any statistical test employed should be justified.
- Source Data should be included to report the data underlying figures according to the guidelines set out in the authorship guidelines on Data Presentation.

2. Captions
Each figure caption should contain the following information, for each panel where they are relevant:
- a specification of the experimental system investigated (eg cell line, species name).
- the assay(s) and method(s) used to carry out the reported observations and measurements.
- an explicit mention of the biological and chemical entity(ies) that are being measured.
- an explicit mention of the biological and chemical entity(ies) that are altered/varied/perturbed in a controlled manner.
- the exact sample size (n) for each experimental group/condition, given as a number, not a range;
- a description of the sample collection allowing the reader to understand whether the samples represent technical or biological replicates (including how many animals, litters, cultures, etc.).
- a statement of how many times the experiment shown was independently replicated in the laboratory.
- definitions of statistical methods and measures:
  - common tests, such as t-test (please specify whether paired vs. unpaired), simple χ2 tests, Wilcoxon and Mann-Whitney tests, can be unambiguously identified by name only, but more complex techniques should be described in the methods section;
  - are tests one-sided or two-sided?
  - are there adjustments for multiple comparisons?
  - exact statistical test results, e.g., P values = x but not P values < x;
  - definition of 'center values' as median or average;
  - definition of error bars as s.d. or s.e.m.

Please complete ALL of the questions below.
Select "Not Applicable" only when the requested information is not relevant for your study.

Materials

Newly Created Materials	Information included in the manuscript?	In which section is the information available? (Reagents and Tools Table, Materials and Methods, Figures, Data Availability Section)
New materials and reagents need to be available; do any restrictions apply?	Not Applicable	

Antibodies	Information included in the manuscript?	In which section is the information available? (Reagents and Tools Table, Materials and Methods, Figures, Data Availability Section)
For **antibodies** provide the following information:<br>- Commercial antibodies: RRID (if possible) or supplier name, catalogue number and or/clone number<br>- Non-commercial: RRID or citation	Yes	Materials and Methods

DNA and RNA sequences	Information included in the manuscript?	In which section is the information available? (Reagents and Tools Table, Materials and Methods, Figures, Data Availability Section)
**Short novel DNA or RNA including primers, probes**: provide the sequences.	Not Applicable	

Cell materials	Information included in the manuscript?	In which section is the information available? (Reagents and Tools Table, Materials and Methods, Figures, Data Availability Section)
**Cell lines:** Provide species information, strain. Provide accession number in repository **OR** supplier name, catalog number, clone number, and/**OR** RRID.	Yes	Material and Methods
**Primary cultures:** Provide species, strain, sex of origin, genetic modification status.	Not Applicable	
Report if the cell lines were recently **authenticated** (e.g., by STR profiling) and tested for mycoplasma contamination.	Not Applicable	

Experimental animals	Information included in the manuscript?	In which section is the information available? (Reagents and Tools Table, Materials and Methods, Figures, Data Availability Section)
**Laboratory animals or Model organisms:** Provide species, strain, sex, age, genetic modification status. Provide accession number in repository **OR** supplier name, catalog number, clone number, **OR** RRID.	Yes	Material and Methods
**Animal observed in or captured from the field:** Provide species, sex, and age where possible.	Not Applicable	
Please detail **housing and husbandry conditions**.	Yes	Material and Methods

Plants and microbes	Information included in the manuscript?	In which section is the information available? (Reagents and Tools Table, Materials and Methods, Figures, Data Availability Section)
**Plants:** provide species and strain, ecotype and cultivar where relevant, unique accession number if available, and source (including location for collected wild specimens).	Not Applicable	
**Microbes:** provide species and strain, unique accession number if available, and source.	Not Applicable	

Human research participants	Information included in the manuscript?	In which section is the information available? (Reagents and Tools Table, Materials and Methods, Figures, Data Availability Section)
If collected and within the bounds of privacy constraints report on age, sex and gender or ethnicity for all study participants.	Not Applicable	

Core facilities	Information included in the manuscript?	In which section is the information available? (Reagents and Tools Table, Materials and Methods, Figures, Data Availability Section)
If your work benefited from core facilities, was their service mentioned in the acknowledgments section?	Yes	Acknowledgment

Design

Study protocol	Information included in the manuscript?	In which section is the information available? (Reagents and Tools Table, Materials and Methods, Figures, Data Availability Section)
If study protocol has been **pre-registered, provide DOI in the manuscript**. For clinical trials, provide the trial registration number **OR** cite DOI.	Not Applicable	
Report the **clinical trial registration number** (at ClinicalTrials.gov or equivalent), where applicable.	Not Applicable	

Laboratory protocol	Information included in the manuscript?	In which section is the information available? (Reagents and Tools Table, Materials and Methods, Figures, Data Availability Section)
Provide DOI OR other citation details if **external detailed step-by-step protocols** are available.	Yes	Material and Methods

Experimental study design and statistics	Information included in the manuscript?	In which section is the information available? (Reagents and Tools Table, Materials and Methods, Figures, Data Availability Section)
Include a statement about **sample size** estimate even if no statistical methods were used.	Yes	
Were any steps taken to minimize the effects of subjective bias when allocating animals/samples to treatment (e.g. **randomization procedure**)? If yes, have they been described?	Not Applicable	
Include a statement about **blinding** even if no blinding was done.	Not Applicable	
Describe **inclusion/exclusion criteria** if samples or animals were excluded from the analysis. Were the criteria pre-established?<br><br>If sample or data points were omitted from analysis, report if this was due to attrition or intentional exclusion and provide justification.	Not Applicable	
For every figure, are **statistical tests** justified as appropriate? Do the data meet the assumptions of the tests (e.g., normal distribution)? Describe any methods used to assess it. Is there an estimate of variation within each group of data? Is the variance similar between the groups that are being statistically compared?	Yes	Material and Methods, Figure Legends

Sample definition and in-laboratory replication	Information included in the manuscript?	In which section is the information available? (Reagents and Tools Table, Materials and Methods, Figures, Data Availability Section)
In the figure legends: state number of times the experiment was **replicated** in laboratory.	Yes	Material and Methods, Figure Legends
In the figure legends: define whether data describe **technical or biological replicates**.	Yes	Figure Legends

Ethics

Ethics	Information included in the manuscript?	In which section is the information available? (Reagents and Tools Table, Materials and Methods, Figures, Data Availability Section)
Studies involving **human participants**: State details of **authority granting ethics approval** (IRB or equivalent committee(s), provide reference number for approval.	Not Applicable	
Studies involving **human participants**: Include a statement confirming that **informed consent** was obtained from all subjects and that the experiments conformed to the principles set out in the WMA Declaration of Helsinki and the Department of Health and Human Services Belmont Report.	Not Applicable	
Studies involving **human participants:** For publication of **patient photos**, include a statement confirming that consent to publish was obtained.	Not Applicable	
Studies involving experimental **animals**: State details of **authority granting ethics approval** (IRB or equivalent committee(s), provide reference number for approval. Include a statement of compliance with ethical regulations.	Yes	Material and Methods
Studies involving **specimen and field samples:** State if relevant **permits** obtained, provide details of authority approving study; if none were required, explain why.	Not Applicable	

Dual Use Research of Concern (DURC)	Information included in the manuscript?	In which section is the information available? (Reagents and Tools Table, Materials and Methods, Figures, Data Availability Section)
Could your study fall under dual use research restrictions? Please check biosecurity documents and list of **select agents and toxins** (CDC): https://www.selectagents.gov/sat/list.htm	Not Applicable	
If you used a select agent, is the security level of the lab appropriate and reported in the manuscript?	Not Applicable	
If a study is subject to dual use research of concern regulations, is the name of the **authority granting approval and reference number** for the regulatory approval provided in the manuscript?	Not Applicable	

Reporting

The MDAR framework recommends adoption of discipline-specific guidelines, established and endorsed through community initiatives. Journals have their own policy about requiring specific guidelines and recommendations to complement MDAR.

Adherence to community standards	Information included in the manuscript?	In which section is the information available? (Reagents and Tools Table, Materials and Methods, Figures, Data Availability Section)
State if relevant guidelines or checklists (e.g., **ICMJE, MIBBI, ARRIVE, PRISMA**) have been followed or provided.	Not Applicable	
For **tumor marker prognostic studies,** we recommend that you follow the **REMARK** reporting guidelines (see link list at top right). See author guidelines, under 'Reporting Guidelines'. Please confirm you have followed these guidelines.	Not Applicable	
For **phase II and III randomized controlled trials**, please refer to the **CONSORT** flow diagram (see link list at top right) and submit the CONSORT checklist (see link list at top right) with your submission. See author guidelines, under 'Reporting Guidelines'. Please confirm you have submitted this list.	Not Applicable	

Data Availability

Data availability	Information included in the manuscript?	In which section is the information available? (Reagents and Tools Table, Materials and Methods, Figures, Data Availability Section)
Have **primary datasets** been deposited according to the journal's guidelines (see 'Data Deposition' section) and the respective accession numbers provided in the Data Availability Section?	Yes	Data Availability
Were **human clinical and genomic datasets** deposited in a public access-controlled repository in accordance to ethical obligations to the patients and to the applicable consent agreement?	Not Applicable	
Are **computational models** that are central and integral to a study available without restrictions in a machine-readable form? Were the relevant accession numbers or links provided?	Not Applicable	
If publicly available data were reused, provide the respective **data citations in the reference list**.	Not Applicable	
